# Protein Signature of Lung Cancer Tissues

**DOI:** 10.1371/journal.pone.0035157

**Published:** 2012-04-11

**Authors:** Michael R. Mehan, Deborah Ayers, Derek Thirstrup, Wei Xiong, Rachel M. Ostroff, Edward N. Brody, Jeffrey J. Walker, Larry Gold, Thale C. Jarvis, Nebojsa Janjic, Geoffrey S. Baird, Sheri K. Wilcox

**Affiliations:** 1 SomaLogic, Inc., Boulder, Colorado, United States of America; 2 Department of Laboratory Medicine, University of Washington, Seattle, Washington, United States of America; 3 Department of Molecular, Cellular, and Developmental Biology, University of Colorado, Boulder, Colorado, United States of America; Ospedale Pediatrico Bambino Gesù, Italy

## Abstract

Lung cancer remains the most common cause of cancer-related mortality. We applied a highly multiplexed proteomic technology (SOMAscan) to compare protein expression signatures of non small-cell lung cancer (NSCLC) tissues with healthy adjacent and distant tissues from surgical resections. In this first report of SOMAscan applied to tissues, we highlight 36 proteins that exhibit the largest expression differences between matched tumor and non-tumor tissues. The concentrations of twenty proteins increased and sixteen decreased in tumor tissue, thirteen of which are novel for NSCLC. NSCLC tissue biomarkers identified here overlap with a core set identified in a large serum-based NSCLC study with SOMAscan. We show that large-scale comparative analysis of protein expression can be used to develop novel histochemical probes. As expected, relative differences in protein expression are greater in tissues than in serum. The combined results from tissue and serum present the most extensive view to date of the complex changes in NSCLC protein expression and provide important implications for diagnosis and treatment.

## Introduction

Progression from healthy state to disease is accompanied by changes in protein expression in affected tissues. Comparative interrogation of the human proteome in healthy and diseased tissues can offer insights into the biology of disease and lead to discovery of new biomarkers for diagnostics, new targets for therapeutic intervention, and identification of patients most likely to benefit from targeted treatment. In particular, new diagnostics for early detection of lung cancer are urgently needed. For the purposes of treatment and prognosis, lung cancer is classified pathologically as either small cell (15%) or non-small cell (85%). Lung cancer is the leading cause of cancer deaths, largely because 84% of cases are diagnosed at an advanced stage, with a five-year survival rate of less than 15% [1]–[3]. Worldwide in 2008, 1.5 million people were diagnosed and 1.3 million died – a survival rate unchanged since 1960 [4]. However, patients diagnosed with NSCLC at an early stage and treated surgically to remove their tumors experience an 86% five-year survival [1], [2].

We recently developed a novel affinity-based proteomic technology for biomarker discovery that currently measures over 1,000 proteins from small sample volumes of plasma or serum (e.g. ∼10 µL of plasma) with low limits of detection (median value of 300 fM), 7 logs of overall dynamic range (∼30 fM – 1 µM, using sample dilution), and 5% median coefficient of variation [5]. This technology, called SOMAscan, is enabled by SOMAmers (Slow Off-rate Modified Aptamers), a new class of protein binding reagents that contain chemically modified nucleotides, which greatly expand the physicochemical diversity of the nucleic acid libraries. Such modifications introduce functional groups that are often found in protein-protein interaction, antibody-antigen interactions, and interactions between small-molecule drugs with their protein targets, but are absent in natural nucleic acids. These modifications are compatible with the SELEX (Systematic Evolution of Ligands by EXponential Enrichment) process used to create SOMAmers as well as standard DNA methods including PCR and hybridization. Overall, the use of these modifications expands the range of possible targets for SELEX, results in improved binding properties, and facilitates selection of SOMAmers with slow dissociation rates [5].

SOMAscan is a highly multiplexed platform for quantitatively measuring proteins in complex matrices such as plasma or serum in which a signature of protein concentrations is transformed into a corresponding DNA signature, which is then quantified on a commercial DNA microarray platform [5]. Briefly, equilibrium binding between a mixture of SOMAmers and proteins is achieved in solution, followed by removal of unbound species by successive bead-based immobilization steps accompanied with extensive washing. High specificity, already an intrinsic feature of SOMAmers, is additionally enhanced with the inclusion of dextran sulfate during binding and washing steps. Dextran sulfate, which like nucleic acids is a polyanion, is effective because cognate SOMAmer-protein complexes are more kinetically stable than non-specific complexes. At the end of the assay, specific SOMAmer-protein complexes remain from which SOMAmers can be eluted under denaturing conditions, hybridized on commercially available microarrays, and directly quantified through a fluorophore covalently coupled to the SOMAmer. In essence, the assay takes advantage of the dual nature of SOMAmers as both folded binding entities with defined shapes and unique nucleic acid sequences recognizable by specific hybridization probes. The utility of this assay has been shown previously in simultaneous measurements of large numbers of proteins ranging from low picomolar to high micromolar concentration in plasma and serum and clinical biomarker studies of chronic kidney disease and lung cancer [5], [6].

## Results

### Proteomic analysis of NSCLC surgical resections

In this report, we performed large-scale protein expression analysis of homogenized lung tissue samples from surgical resections obtained from eight non-small cell lung cancer (NSCLC) patients. All NSCLC patients were smokers, ranging in age from 47 to 75 years old and diagnosed with pathology-confirmed NSCLC stages IA through IIIB (Table 1). We obtained three samples from each resection: tumor tissue sample, adjacent non-tumor tissue (within 1 cm of the tumor) and distant uninvolved lung tissue (furthest edge of the resection from the tumor). Care was taken to preserve the integrity of the tissue, with all samples being frozen within 5–10 minutes of excision. Total protein concentration was adjusted and normalized in each homogenate for proteomic profiling followed by analysis on our biomarker discovery array to measure the concentrations of 820 human proteins as recently described [5].

**Table 1:** Patient demographics, resection location and tumor types for the eight NSCLC analyzed samples.

Age	Sex	Smoking History	Location	Stage	Tissue Dx
47	F	Smoker	Left Upper Lobe	pT3pN1pMx stage IIIA	Poorly differentiated non-small cell CA with focal squamous differentiation
73	F	Smoker	Left Lower Lobe	pT2pN0pMx stage IB	Poorly differentiated squamous cell carcinoma
48	M	Smoker	Right Upper Lobe	pT2pN1pMx stage IIIA	Poorly differentiated squamous cell carcinoma
60	F	Smoker	Left Upper Lobe	T4 N1 M0 stage IIIB - note T4 distinction based on clinical lung collapse; tumor was pT2 by size criteria	Poorly differentiated squamous cell carcinoma
51	F	Smoker	Right Upper Lobe	pT2pN0pMx stage IB	Moderately differentiated adenocarcinoma
71	F	Smoker	Right Upper Lobe	pT2pN0pMx stage IB	Well differentiated adenocarcinoma
75	F	Smoker	Right Lower Lobe	pT1N0Mx Stage IA	Well differentiated adenocarcinoma
73	M	Smoker	Left Upper Lobe	pT1bN0Mx Stage IA	Atypical carcinoid tumor (i.e. neuroendocrine, IHC positive for chromogranin)

**Figure 1:**
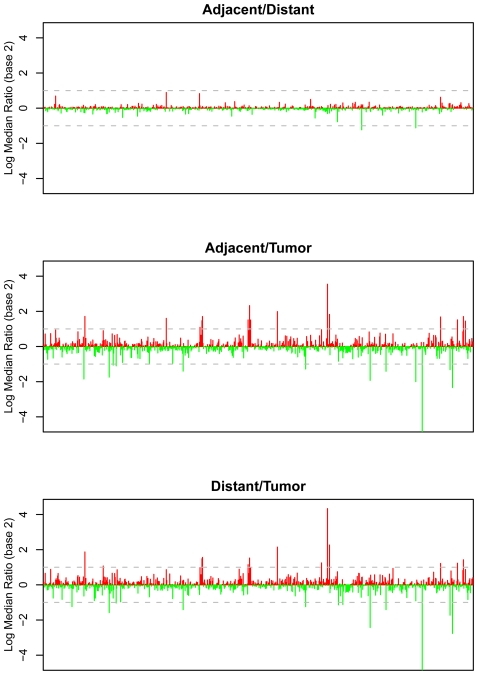
Relative changes in protein expression for 820 proteins from eight NSCLC resection samples. Signal differences between adjacent and distant tissue (panel A), tumor and adjacent tissue (panel B) and tumor and distant tissue (panel C) are expressed as log_2_ median ratios. The dotted line represents two-fold change (log_2_ = 1).

These protein concentration measurements, expressed as relative fluorescence units (RFU), allow large-scale comparisons of protein signatures among samples (Fig. 1). We first compared the protein expression levels between the adjacent and distant tissue samples for each patient (Fig. 1A). Overall, the signals generated by most analytes were similar in adjacent and distant tissue. In this comparison, only one analyte (fibrinogen) exhibited more than a two-fold difference between the two control samples. Fibrinogen concentration was higher in adjacent non-tumor tissue than distant non-tumor tissue. Fibrinogen is the soluble precursor of fibrin, which is converted by thrombin during coagulation. Fibrin deposits occur within adjacent stroma of most tumors, primarily in the extracellular matrix (ECM) where fibrin and other ECM proteins promote and support tumor growth processes including, cell proliferation, adhesion, invasion, migration, and angiogenesis [7].

**Figure 2:**
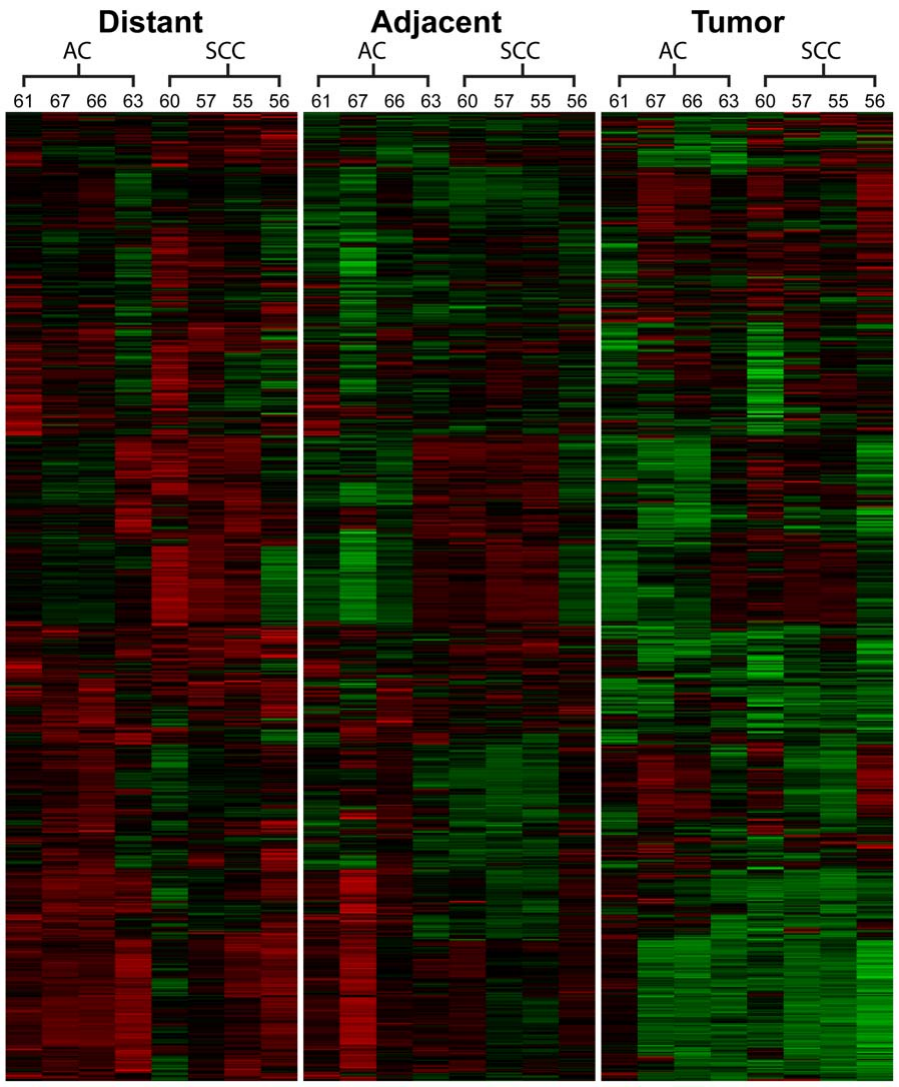
Heat map of protein levels in tumor tissue samples. The samples are displayed in columns and separated into distant non-tumor, adjacent non-tumor, and tumor tissue. Within each tissue type, the samples are separated into adenocarcinomas (AC) or squamous cell carcinomas (SCC). The numbers above each column correspond to patient codes. The proteins are displayed in rows and were ordered using hierarchical clustering.

In contrast, comparison of tumor tissues with non-tumor tissue (adjacent or distant) identified 11 (1.3%) proteins with greater than four-fold differences and 53 (6.1%) proteins with greater than two-fold differences (Figs. 1B and 1C). The remaining (93.9%) proteins showed relatively small differences between tumor and non-tumor tissue. Some proteins were substantially suppressed while others were elevated in tumor tissues compared to adjacent or distant tissues. Differential expression of proteins between adjacent and tumor tissue, or between distal and tumor tissue, was similar overall. Changes in between tumor and distal tissue were generally somewhat larger compared to tumor and adjacent tissue (Fig. 1), which demonstrates that most observed protein changes are specific to the local tumor environment. Figure 2 shows a heat map depiction of the results. There was a trend of protein changes reflecting pathologic stage, which may indicate that protein expression correlates with disease burden. Given the small sample size, correlations with histological classification could not be decoupled from stage.

### Biomarker identification

**Figure 3:**
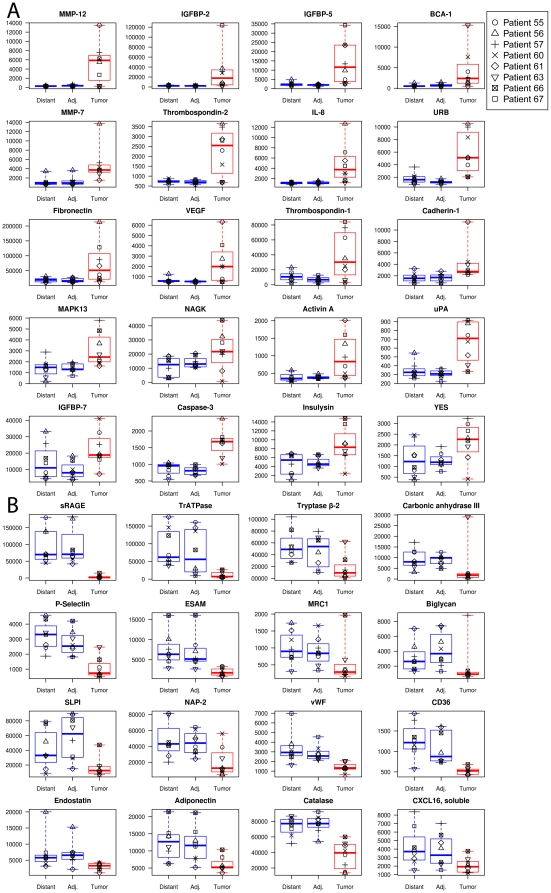
Box plots of SOMAmer signals in the tissue homogenates. Proteins with increased (panel A) or decreased (panel B) levels in tumor tissue compared with adjacent or distal tissue (panel A) from eight NSCLC samples used in this study. Each individual is indicated with a different symbol. The horizontal lines of each box correspond to the first, second, and third quartiles (25%/50%/75%) and the whiskers correspond to the maximum and minimum values.

**Figure 4:**
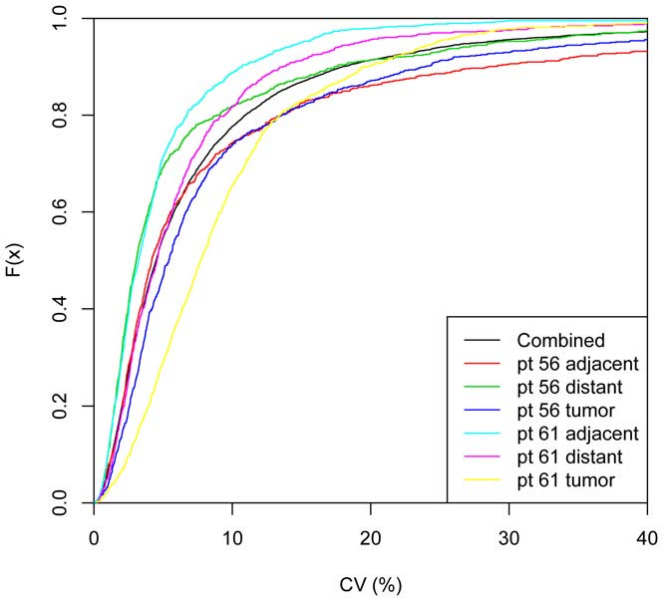
Plot of the cumulative density function (CDF) for the coefficient of variation (CV) between triplicate samples. The tumor, adjacent non-tumor, and distant non-tumor tissue resections were sampled, extracted, and analyzed with the SOMAscan proteomic assay in triplicate for two individuals in the study. The median CV for all 6 triplicates was 4.5% (black line).

**Figure 5:**
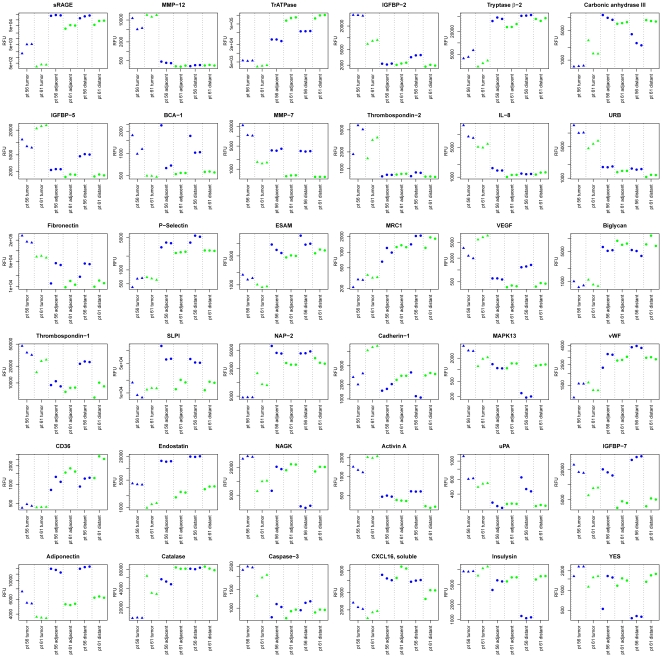
Plots of triplicate samples for the 36 analytes with the largest mean fold-change in protein expression between tumor and non-tumor tissue samples (Table 2). The tumor, adjacent non-tumor, and distant non-tumor tissue resections were sampled, extracted, and analyzed with the SOMAscan proteomics assay in triplicate for two individuals (patients 56 and 61) in the study. The samples are colored by individual and the tumor samples are highlighted as triangles. The y-axis is on a log scale.

To identify potential NSCLC tissue biomarkers, we looked for analytes with the largest change in protein expression levels between tumor, adjacent, and distant tissue samples. Here we highlight thirty-six proteins with the largest mean fold-change in protein expression between tumor and non-tumor tissue samples (Fig. 3, Table 2). We tested the significance of these changes with the Mann Whitney test and required a p-value of 0.05 after correcting for multiple tests (false discovery rate cutoff of q<0.05). Although the number of samples we used for this study was relatively small, the study consisted of paired tumor and non-tumor tissue samples from each individual. This provides more power to identify changes within an individual and eliminates the population variance associated with cross-sectional study designs. The availability of appropriately chosen reference samples is increasingly recognized as a crucially important component in biomarker discovery research [8]–[10]. Finally, we assessed reproducibility of this new method by analyzing triplicate samples of tumor and non-tumor tissue resections for two subjects in this study and found a 4.5% median CV between triplicate measurements for the 820 proteins measured (Fig. 4) and Spearman correlation coefficients >0.99 (partially inflated by the large RFU range measured). Triplicate measurements for the 36 proteins with largest mean-fold differences between tumor and non-tumor tissue are plotted in Fig. 5.

**Table 2:** SOMAscan protein expression differences expressed as log_2_ ratio of the signal in tumor to non-tumor tissue samples for proteins with the largest mean-fold change.

Protein Target	Adjacent	Distant	NSCLC Tissue Expression Changes Reported in Literature
Activin A	1.16	0.88	Protein [39]; Gene [39]–[41]
Adiponectin	−0.99	−1.00	None
BCA-1	1.91	2.24	None
Biglycan	−1.88	−1.14	None
Cadherin-1	1.33	1.21	Protein [42]–[45]
Carbonic anhydrase III	−2.22	−2.13	None
Caspase-3	0.95	0.98	Protein [46], [47]; Gene [41], [48]
Catalase	−1.03	−0.93	Protein [49], [50]; Gene [50], [51]
CD36	−0.91	−1.24	Gene [52]
CXCL16, soluble	−0.93	−1.00	None
Endostatin	−1.03	−1.12	Protein [13], [14], [53]
ESAM	−1.52	−1.81	Protein [54]
Fibronectin	1.92	1.58	None
IGFBP-2	2.42	2.58	Gene [55], [56]
IGFBP-5	2.45	1.77	Gene [56], [57]
IGFBP-7	1.52	0.47	Protein [58], [59]
IL-8	1.80	1.76	Protein [60]; Gene [52], [60], [61]
Insulysin	0.78	1.10	None
MAPK13	1.28	0.89	None
MMP-7	1.91	2.11	Protein [26], [62]; Gene [52], [63]
MMP-12	3.53	4.19	Protein[26], [27]; Gene [27], [41], [51], [64]
MRC1	−1.36	−1.96	None
NAGK	0.83	1.24	None
NAP-2	−1.11	−1.44	Gene [41]
P-Selectin	−1.57	−1.78	None
SLPI	−1.77	−0.85	Gene [51]
sRAGE	−5.77	−5.44	Protein [25], [65]; Gene [25], [41], [51], [65]
Thrombospondin-1	1.70	1.23	Protein [20]; Gene [40], [52]
Thrombospondin -2	1.80	1.93	Protein[66]; Gene [40], [52], [66]
TrATPase	−2.26	−2.87	None
Tryptase β-2	−2.68	−2.24	Protein [67]
uPA	1.08	0.94	Protein [68]; Gene [52], [64], [68], [69]
URB	2.11	1.41	None
VEGF	1.88	1.34	Protein [12], [14]; Gene [15], [41], [51]
vWF	−0.98	−1.18	Gene [41], [51]
YES	0.85	1.02	Gene [70], [71]

High-content proteomic analysis of biological samples enabled by our multiplexed assay allows unbiased discovery of disease-related proteins. To date, we have conducted several blood-based clinical biomarker studies of human diseases, including lung cancer [6] and chronic kidney disease [5]. These studies have identified novel potential disease biomarkers as well as biomarkers that have been reported previously. The current study follows this trend. About one-third (13/36) of the potential NSCLC tissue biomarkers identified here are novel, to the best of our knowledge. The remaining two-thirds (23/36) have been reported previously as differentially expressed proteins or genes in NSCLC tumor tissue (Table 2). Novelty was determined by performing literature searches in Pubmed and on the internet using the potential biomarkers' gene names and protein aliases as identified by UniProt.

The potential biomarkers can be classified broadly into four biological processes associated with important hallmarks of tumor biology [11] as shown in Table 3: 1) angiogenesis, 2) growth and metabolism, 3) inflammation and apoptosis, and 4) invasion and metastasis. Admittedly, these are convenient but inexact classifications that approximate a highly complex and dynamic system in which these molecules often play multiple and nuanced roles. Therefore, the specific state of a given system ultimately affects the expression and function of any particular molecule. Our understanding of the biological underpinnings of these systems is far from complete. With the SOMAscan platform, we are beginning to explore the quantitative expression of large numbers of proteins in various tissues and disease processes. These data provide new coordinates to help map the dynamics of these systems, which in turn will provide a more complete understanding of the biology of this disease. The results from the current study provide a new perspective on NSCLC tumor biology, with both familiar and new elements.

**Table 3:** Categorization of NSCLC tissue biomarkers into biological major processes.

Angiogenesis	Growth and Metabolism	Inflammation & Apoptosis	Invasion, Metastasis (ECM)
VEGF	Adiponectin*	Activin A	Biglycan*
Endostatin	Carbonic anhydrase III*	BCA-1*	Cadherin-1
Thrombospondin-1	IGFBP-2	Catalase	CD36
Thrombospondin-2	IGFBP-5	CXCL16, soluble*	ESAM
	IGFBP-7	IL-8	Fibronectin*
	Insulysin*	MRC1*	MMP-7
	NAGK*	NAP-2	MMP-12
	TrATPase*	sRAGE	P-Selectin*
	Tryptase b-2	SLPI	URB*
	MAPK13*	uPA	vWF
	Caspase-3	Thrombospondin-1	
	Thrombospondin-2		
	YES		

* Novel NSCLC Biomarker.

### Angiogenesis

Angiogenesis drives growth of new blood vessels to support tumor growth and metabolism. The regulation of angiogenesis is a complex biological phenomenon controlled by both positive and negative signals [11]. Among the potential NSCLC tissue biomarkers identified in this study (Fig. 3) were well known positive and negative angiogenesis regulators, all of which have been observed previously in NSCLC tumor tissue [12]–[16]. These include the prototypic angiogenesis inducer VEGF and inhibitors endostatin and thrombospondin-1 (TSP-1). VEGF is a powerful growth factor that promotes new blood vessel growth; VEGF was strongly up-regulated in NSCLC tumor tissue, consistent with previous observations [12], including our study of serum samples from NSCLC patients [6]. It is worth noting that VEGF was originally discovered as tumor cell-secreted vascular permeability factor (VPF) that increased the leakiness of tumor-associated blood vessels to large molecules, such as fibrinogen, that are normally confined to plasma [17]. This activity may have profound effects on the composition of proteins associated with tumor tissue. Endostatin is a proteolytic fragment of collagen XVIII and a strong inhibitor of endothelial cell proliferation and angiogenesis [13]. TSP-1 and the related TSP-2 were substantially up-regulated in NSCLC tumor tissue. TSP-1 and TSP-2 are extracellular matrix proteins with complex, context-dependent effects modulated through a variety of interactions with cell-surface receptors, growth factors, cytokines, matrix metalloproteinases, and other molecules. Archetypically in model systems, TSP-1 and TSP-2 inhibit angiogenesis by inhibiting endothelial cell proliferation through the CD47 receptor (not measured in this study) and inducing endothelial cell apoptosis through the CD36 receptor. There is also evidence for proangiogenic influences for TSP-1 and TSP-2 [18]. Finally, reported TSP-1 and TSP-2 relative and absolute expression levels in NSCLC tissue vary [16], [19]–[21] likely due to their complex functions. In our study, we also found that CD36 was down-regulated in NSCLC tumor tissue, which could indicate an adaptation of tumor cells reduce sensitivity to TSP-1 and TSP-2-mediated apoptosis.

### Growth and Metabolism

Ten of the potential NSCLC biomarkers we identified are associated with growth and metabolism functions. Half of these biomarkers are involved in the complex hormonal regulation of cellular growth and energy metabolism. Three insulin-like growth factor binding proteins (IGFBPs), which modulate the activity of insulin-like growth factors (IGFs), were up-regulated in NSCLC tumors (IGFBP-2, -5, and -7). Several reports have qualitatively assessed IGFBP-2, -5, and -7 in NSCLC (Table 2) and suggest higher expression in NSCLC tissue than in normal tissue. Insulin and IGFs act as hormones that strongly influence cellular growth, metabolism, and survival. Cancer cells are often dependent on these molecules for growth and proliferation [11]. IGFBP-2 has also been associated with an anti-apoptotic effect via caspase-3 [22]. These hormones are in turn degraded by insulysin [23], whose concentration was higher in NSCLC tumor tissue. The hormone adiponectin controls lipid metabolism and insulin sensitivity, and we found adiponectin down-regulated in NSCLC tumors. The remaining five biomarkers, carbonic anhydrase III, NAGK, TrATPase, tryptase β-2, and MAPK13, are all enzymes with known roles in cellular metabolism (Table 3).

### Inflammation and Apoptosis

Inflammation and apoptosis are hallmarks of cancer biology, and we find a number of potential biomarkers associated with these processes that have been associated previously with NSCLC (Table 2). We found caspase-3 concentrations higher in NSCLC tumor tissue. Caspase-3 has been associated with metastasis [24]. Another notable example is soluble receptor for advanced glycation end-products sRAGE, which has been reported to be dramatically down-regulated in NSCLC tissue [21], [25]. This finding is consistent with our measurement, in which sRAGE had the largest observed change for proteins that are lower in tumor than in non-malignant tissue. One hypothesis is that RAGE plays a role in epithelial organization, and decreased levels of RAGE in lung tumors may contribute to loss of epithelial tissue structure, potentially leading to malignant transformation [25]. Several chemokines, such as BCA-1, CXCL16, IL-8, and NAP-2, are altered in our study, consistent with the hypothesis that invasion of tumors with cells from the innate and adaptive arms of the immune system provide bioactive molecules that affect proliferative and angiogenic signals [11].

### Invasion and Metastasis

The largest group of potential biomarkers contains proteins that function in cell-cell and cell-matrix interactions and are involved in invasion and metastasis. Many have been previously reported to be associated with NSCLC. Most notable are two of the matrix metalloproteases, MMP-7 and MMP-12, which contribute to proteolytic degradation of extracellular matrix components and processing of substrates such as growth factors. For example, the major substrate for MMP-12 is elastin. Such processes are well known to play a role in creating tumor microenvironments. We observed MMP-7 and MMP-12 up-regulated in NSCLC tissue, which is consistent with similar study that used antibody-based measurements [26]. The over-expression of MMP-7 and MMP-12 has been associated with poor prognosis in NSCLC [26]. MMP-12 levels have been correlated with local recurrence and metastatic disease [26]. It is interesting to note that two of the eight subjects studied had normal levels of MMP-12, whereas the other six had 15–50-fold elevation of MMP-12 in tumor tissue compared to non-tumor tissue.

### SOMAmers as histochemistry probes to NSCLC biomarkers

**Figure 6:**
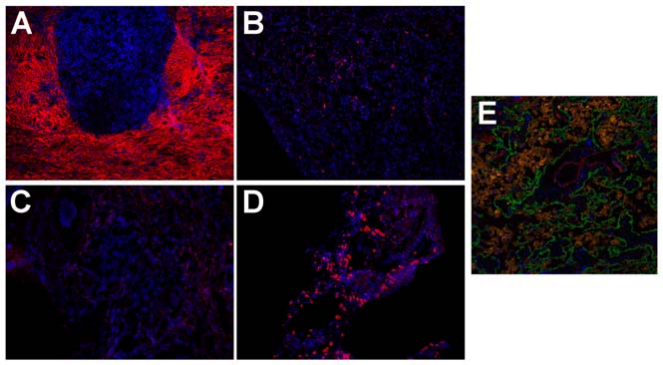
SOMAmer histochemistry on frozen tissue sections for selected biomarkers detected in this study. (A) Thrombospondin-2 SOMAmer (red) staining the fibrocollagenous matrix surrounding a tumor nest. (B) Corresponding normal lung specimen stained with Thrombospondin-2 SOMAmer (red). (C) Macrophage mannose receptor SOMAmer (red) staining scattered macrophages in a lung adenocarcinoma. (D) Macrophage Mannose Receptor SOMAmer (red) staining numerous alveolar macrophages in a section of normal lung parenchyma. (E) Multicolor image highlighting the cytomorphologic distribution of macrophage mannose receptor SOMAmer staining: Green = Cytokeratin (AE1/AE3 antibody), Red = CD31 (EP3095 Antibody), and Orange = SOMAmer. All nuclei in this figure are counterstained with DAPI.

**Figure 7:**
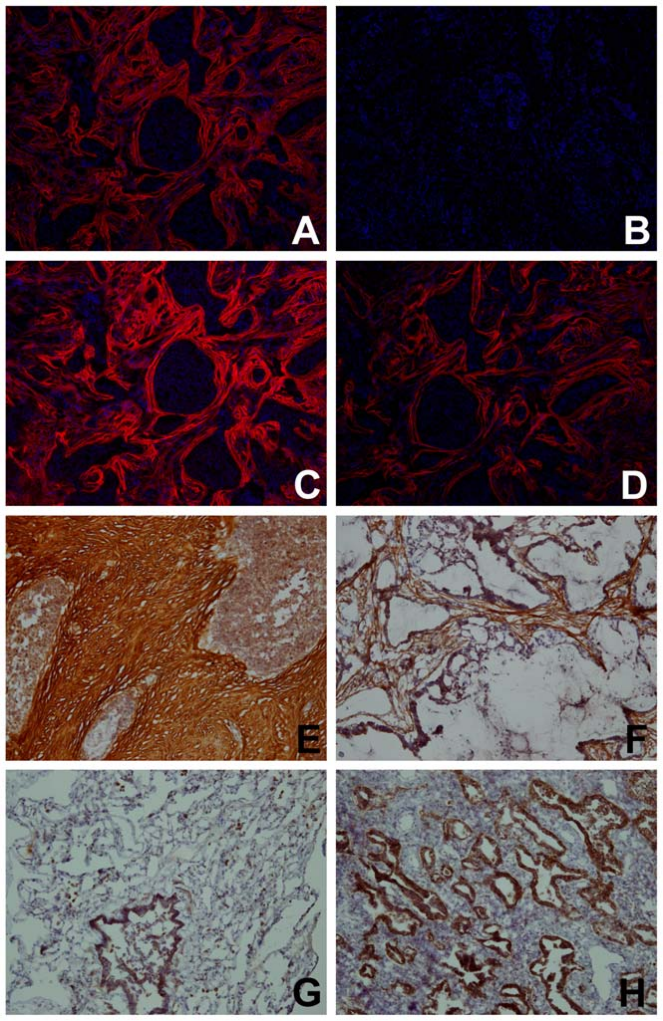
Thrombospondin-2 (TSP-2) histochemical identification in tissue samples. TSP-2 is identified in serial frozen sections of a single lung carcinoma specimen by (A) a home-made rabbit polyclonal TSP-2 polyclonal antibody, (B) the pre-immune serum from rabbits used to make the home-made polyclonal antibody, (C) a commercial (Novus) rabbit polyclonal TSP-2 antibody, and (D) the TSP-2 SOMAmer. The TSP-2 SOMAmer was used to stain frozen sections of normal and malignant lung tissue, with standard Avidin-Biotin-Peroxidase color development, to demonstrate different morphologic distributions: (E) Strong staining of the fibrotic stroma surrounding tumor nests, with minimal cytosolic staining of carcinoma cells, (F) Strong staining of the fibrotic stroma surrounding tumor nests in a mucinous adenocarcinoma, with no significant staining of the carcinoma cells, (G) normal lung tissue, showing strong cytosolic staining of bronchial epithelium and scattered alveolar macrophages, and (H) strong cytosolic staining of an adenocarcinoma, with no significant staining of the non-fibrotic, predominantly inflammatory stroma.

Understanding the differences in protein expression between tumor and non-tumor tissues can identify novel histochemistry targets. This approach was used previously with MMP-12 and others [27]. Such probes can enable more precise molecular characterization of tumors and their effects on the surrounding stroma. We have previously demonstrated that fluorophore-labeled SOMAmers confer rapid and selective histochemical staining in frozen tissue sections [28]. Here we examined tissue staining by several of the SOMAmers that were identified as biomarkers in our analysis of tissue homogenates. Frozen tissue sections were cut from the same tumor resections used for biomarker discovery. For example, TSP-2 staining with a fluorophore-labeled SOMAmer in tumor tissue was striking and localized predominantly in areas of fibrous stromal scarring (Fig. 6A), but such staining was largely absent in normal tissue (Fig. 6B). This is consistent with the reported role of TSP-2 in matrix modulation [18], [29]. In contrast, in normal lung tissue, the macrophage mannose receptor (MRC1) SOMAmer staining localized to the surface of alveolar macrophages (Fig. 6D) as expected for this target [30]. Tumor tissue samples, which lack alveoli, showed little MRC1 staining (Fig. 6C). Figure 6E demonstrates MRC1 SOMAmer staining performed concurrently with antibody-based immunofluorescence for other targets (cytokeratins and CD31), indicating the feasibility of multiplexing SOMAmer and antibody reagents in histologic studies. Tissue staining with SOMAmers was thus consistent with the homogenate profiles, in which TSP-2 was elevated and MRC1 was decreased in tumor versus healthy tissue. We confirmed the SOMAmer staining patterns of TSP-2 and MRC1 with antibodies Figure 7. Congruence between histochemical staining results with the direction of change in protein expression between tumor and healthy tissue homogenates provides additional evidence that the identified biomarkers are associated with disease. Large-scale proteomic comparison between tissues described here is also a powerful method for identifying novel histochemical probes.

**Figure 8:**
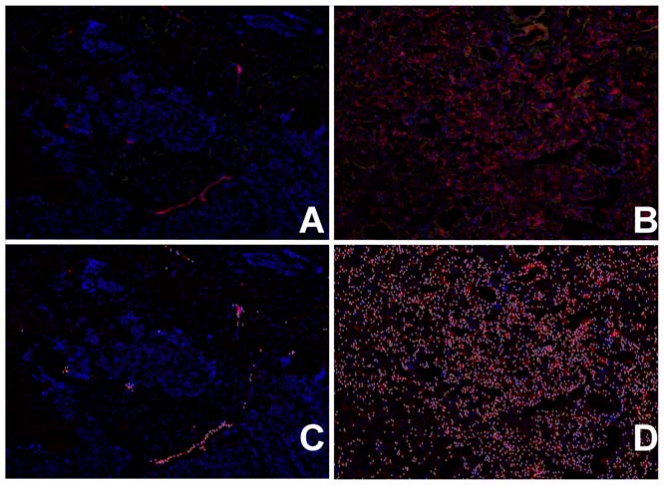
ESAM histochemical staining in tissue samples. ESAM staining is shown in lung tumor (A,C) and normal lung (B,D) distant from the tumor. Endothelial cells are visibly more abundant in the normal lung section, consistent with the high vascularity of normal lung. Raw images are shown in A and C, with ESAM-positive cells identified by the CellProfiler algorithm marked with a “1” in images B and D.

Some general caveats related to discovery of potential NSCLC biomarkers are worth noting. First, the fact that a protein is associated with tumor tissue need not mean that it is specific for tumor tissue. For example, inflammation, extracellular matrix remodeling, hypoxia, and tissue necrosis accompanies tumor progression but also many other non-malignant conditions such as injury, wound healing or infection. Second, biomarkers we identified could reflect a difference in the ratio of cell types that constitute a tumor sample compared to that of the normal lung tissue. For example, if tumor tissue consists of cancer cell overgrowth, some of the biomarkers are expected to be specific for that cell type (in this case, epithelial cells), transformed or not. Similarly, if a tumor tissue sample is either more or less vascularized than the surrounding normal tissue, a change in the expression of endothelial cell-specific proteins may be observed. Indeed, we observed significantly lower concentrations of ESAM, a protein specific to endothelial cells, compared to matched, non-tumor tissue. We confirmed this histochemically, as shown in Figure 8, where we measured 35-fold more ESAM-positive endothelial cells in distant non-tumor tissue compared to tumor tissue. Finally, SOMAmers, like all affinity reagents, bind and recognize specific epitopes of target proteins generally in a conformation-dependent manner, and any particular measurement reflects the availability of that epitope.

### Comparison of NSCLC tissue and serum biomarkers

We have recently completed a NSCLC study [6] in which we analyzed 1,326 serum samples from four independent clinical study centers using the same proteomic platform and a protein menu nearly identical to the that used for tissue (813/820 proteins ). The study included patients diagnosed with pathologic or clinical stage I–III NSCLC and a control population with a history of long-term tobacco use, including active smokers and ex-smokers with at least 10 pack-years of cigarette smoking. Taking extensive precautions to account for pre-analytic variables, we identified 44 candidate biomarkers, and developed a 12-protein panel that distinguished NSCLC from controls with 91% sensitivity and 84% specificity in a training set, and 89% sensitivity and 83% specificity in a blinded, independent verification set. The availability of this database allows us to compare changes in protein expression in tissue and serum from NSCLC patients.

**Figure 9:**
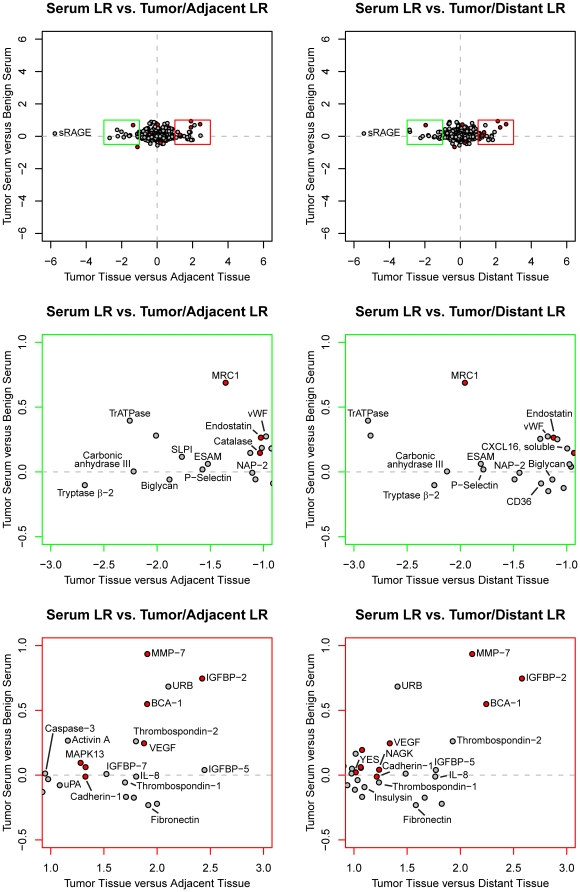
Changes in protein expression in NSCLC tissue compared to serum. The top two panels show the log2 ratio (LR) derived from serum samples versus log ratios derived from adjacent tissue and distant tissue, respectively. The bottom four panels feature zoomed portions of plots above, indicated by the color of the plot (green for decreased and red for increased expression compared to non-tumor tissue). Analytes shown in Figure 2 have been labeled and analytes mentioned in the publication on the serum samples are shown in filled red symbols red.

While the methodology used to analyze the protein profiles in samples from these two NSCLC studies is the same, some significant differences are worth noting. First, serum pre-analytic variability was observed between study centers, perhaps masking some cancer biomarkers [6]. Second, in the NSCLC tissue study reported here, each tumor sample has its own control tissue (adjacent and distant non-tumor), whereas the larger NSCLC serum study by necessity is composed of case and control samples from different individuals. Nevertheless, differential expression of proteins in sera of NSCLC patients relative to cancer-free controls compared with that of NSCLC tissue samples yields useful insights (Fig. 9, Table 4). The most striking observation is that relative changes in protein expression are greater in tissues than in serum. This result could be expected since tumor tissue is the source of the changes in protein expression that is then, even if fully released into circulation, diluted many-fold into total volume of blood. This trend is evident in the elongated distribution of data points along the x-axis in Figure 9 in which axes are drawn on the same scale to illustrate this point. Eleven of the analytes shown in Figure 3 as altered in tumor tissue were also differentially expressed in sera from NSCLC patients vs. controls (filled red circles in Fig. 9). It is worth noting that our published NSCLC serum study [6] did not measure MMP-12, which this study identified as a top tissue biomarker. In subsequent NSCLC serum studies, MMP-12 was measured and we found it was also a top serum biomarker with a KS-distance of 0.42 (Table 2). This suggests that elevated serum MMP-12 directly reflects NSCLC tumor biology. Most of the other biomarkers common to tissue and serum also change in the same direction, but a few do not. Local concentrations of proteins in a tissue homogenate clearly need not correlate with circulating levels of the proteins and inverse correlations may provide clues regarding the redistribution of certain biomarkers in diseased versus normal tissues.

**Table 4:** List of potential NSCLC biomarkers identified from serum and tissue samples.

#	Protein Name	NSCLC Serum	NSCLC Tissue
1	Activin A		Up
2	Adiponectin		Down
3	AMPM2	Up	
4	**BCA-1**	Up	Up
5	Biglycan		Down
6	BMP-1	Down	
7	C1s	Up	
8	C9	Up	
9	**Cadherin-1**	Up	Up
10	Calpain I	Up	
11	Carbonic anhydrase III		Down
12	Caspase-3		Up
13	**Catalase**	Up	Down
14	CD30 Ligand	Up	
15	CD36		Down
16	CDK5/p35	Up	
17	CK-MB	Down	
18	Contactin-5	Down	
19	CXCL16, soluble		Down
20	**Endostatin**	Up	Down
21	ERBB1	Down	
22	ESAM		Down
23	FGF-17	Up	
24	Fibronectin		Up
25	FYN	Up	
26	HSP 90a	Up	
27	HSP 90b	Up	
28	**IGFBP-2**	Up	Up
29	IGFBP-5		Up
30	IGFBP-7		Up
31	IL-15 Ra	Up	
32	IL-17B	UP	
33	IL-8		Up
34	IMB1	Up	
35	Insulysin		Up
36	Kallikrein 7	Down	
37	KPCI	Up	
38	LDH-H 1	Up	
39	LGMN	Up	
40	LRIG3	Down	
41	**MAPK13**	Up	Up
42	MEK1	Up	
43	Midkine	Up	
44	MIP-5	Up	
45	**MMP-12**	Up	Up
46	**MMP-7**	Up	Up
47	**MRC1**	Up	Down
48	NACA	Up	
49	**NAGK**	Up	Up
50	NAP-2		Down
51	PARC	Up	
52	P-Selectin		Down
53	PTN	Up	
54	Renin	Up	
55	RGM-C	Down	
56	SCF sR	Down	
57	SLPI		Down
58	sL-Selectin	Down	
59	sRAGE		Down
60	Thrombospondin-1		Up
61	Thrombospondin-2		Up
62	TrATPase		Down
63	Tryptase β-2		Down
64	Ubiquitin+1	Up	
65	uPA		Up
66	URB		Up
67	**VEGF**	Up	Up
68	vWF		Down
69	**YES**	Up	Up

Proteins identified in both studies are shown in boldface font.

## Discussion

The discovery of novel biomarkers with demonstrable diagnostic or clinical utility has been a considerable challenge in recent years [8]. The reasons for this include: the omnipresence of pre-analytical and analytical artifacts, unavailability of suitable healthy-state controls, issues related to study designs, and the difficulty of detecting small changes in protein levels at very low concentrations. This challenge is especially pronounced with cancer biomarkers where the objective is often to find biomarkers of a tiny malignancy in the blood of a relatively large human body at an early stage.

The recently completed National Lung Screening Trial (NLST) reported a significant mortality benefit of screening for NSCLC with low dose CT and detecting early stage disease in a high risk population [31]. However, the high false positive rate results in a low (4%) positive predictive value (PPV) and potential harm from over diagnosis and unnecessary treatment. Complementary biomarkers that would either identify individuals who would most benefit from CT screening or improve the PPV value of imaging are in the research phase but not are yet in clinical use. Both miRNA and proteomic signatures in tissue and plasma have been reported [32], [33]. Advances in understanding the molecular origins of NSCLC are beginning to guide the development of targeted therapies [34]. As reported by Sequist, approximately half of NSCLC tumors have known driver mutations, 22% of which are candidates for molecular targeted therapy [35].

One way to improve the chances of discovering true cancer biomarkers is to measure protein concentrations in both the source of the disease, such as tumor tissue, as well as from the circulation. Such combined results can support the validity of potential biomarkers and separate them from experimental artifacts. In this report, we have demonstrated that this is possible with our highly multiplexed and sensitive proteomic assay. We have shown that tissues, like plasma or serum, are also amenable to SOMAscan, and the resulting comparative analysis of protein expression in NSCLC tumor tissues with surrounding healthy lung tissues offers a complement to our existing dataset of potential NSCLC biomarkers identified from serum samples. In our case, one third of the thirty-six tissue biomarkers reported here (BCA-1, cadherin-1, catalase, endostatin, IGFBP-2, MRC1, MMP-7, MAPK-13, NAGK, VEGF and YES) have been previously identified in serum [6]. Taken together, these data contribute to further understanding of the complexity of changes accompanying NSCLC and provide us with additional potential biomarkers for the early detection of this deadly disease.

## Materials and Methods

This work was performed on deidentified samples that would have been disposed of if not used for research. This research thus qualified as a minimal risk to patients and their privacy, and approval for the use of these specimens with a waiver of consent was granted by the University of Washington's Institutional Review Board.

### Homogenate Preparation

All tissue samples for proteomic analysis were obtained by freezing the tissue within 5–10 minutes of excision during surgery and after placing the tissues in OCT medium (10.24% polyvinyl alcohol, 4.26% polyethylene glycol, and 85.5% non-reactive ingredients). Three samples were obtained from each resection: tumor tissue sample, adjacent healthy tissue (within 1 cm of the tumor) and distant uninvolved lung tissue. While keeping the samples constantly frozen, five 10-µm thick sections were cut, trimmed of excess OCT from around the tissue, and placed into frozen 1.5 mL microfuge tubes. Following the addition of 200 µl homogenization buffer (40 mM HEPES, 125 mM NaCl, 5 mM KCl at pH 7.5 plus HALT protease inhibitor cocktail (Pierce)), the samples were homogenized in the microfuge tubes on ice with rotary pestle for 30 seconds, until no tissue fragments were visible. The samples were then spun in a centrifuge at 21,000×g for 10 minutes and filtered through a 0.2 µm multi-well plate filter into a sterile multi-well plate. Five µl aliquots were taken for micro-BCA protein assay (Pierce) and the rest of the sample was stored frozen and sealed in 96 well plates at −70°C.

### Proteomic Profiling

Sample total protein was adjusted to16 µg/mL in SB17T buffer (40 mM HEPES, 125 mM NaCl, 5 mM KCl, 5 mM MgCl_2_, 1 mM EDTA, 0.05% Tween-20 at pH 7.5) for proteomic profiling. Samples prepared in this manner were analyzed on the SomaLogic biomarker discovery assay using Agilent slide read-out that measures 820 human proteins as described previously [5]. The proteins against which the SOMAmers included in this assay were selected are given in Table S1. Briefly, this assay uses SOMAmers to transform protein concentration into a corresponding DNA concentration through a series of steps involving affinity binding and capture of biotin onto streptavidin beads. The final DNA concentration is measured as relative fluorescence units (RFU) from the fluorescent SOMAmer hybridized to a complementary probe on an Agilent array.

Since each of the eight tumors in the study varies in stage and histology, we did not assume that the RFU measurements from the tissue resections were normally distributed. Therefore, we tested the significance of differential expression between the eight tumor tissues and the sixteen healthy surrounding tissues using a non-parametric test, specifically the Mann-Whitney rank sum test. We chose to combine the two healthy tissue groups because this study contains a small number of total samples.

A separate two-tailed Mann-Whitney test was performed for each of the 820 human proteins measured by the SOMAscan platform, so we corrected for multiple comparisons by applying a false discovery rate (FDR) correction [36] and enforced a q-value threshold of 0.05 for all analytes.

Due to the low number of samples and the non-parametric nature of the Mann-Whitney test, we identified many significant analytes that were only differentially expressed by a very small magnitude. Therefore, we sorted the set of significantly differentially expressed analytes by their log ratios between the tumor samples and the healthy samples. For this paper, we chose to highlight the 36 analytes with the highest log ratio within the set of significantly differentially expressed analytes.

### SOMAmer staining method

5 µm frozen tissue sections were immediately placed onto a charged slide (Superfrost Plus), and the slide was then immersed in a fixative solution of 100% ethanol (P-Selectin & ESAM) or acetone (all other SOMAmers) for at least 30 minutes. OCT medium was removed from slides by a 2-minute rinse in deionized water, followed by a 2 to 5 minutes rinse in selection buffer SB-T (40 mM Na-HEPES, pH 7.5, 52 mM NaCl, 5 mM KCl, 5 mM MgCl_2_, 0.05% Tween-20). 200 nM SOMAmer solutions made up in SB-T supplemented with 1 mM dextran sulfate (DS) were applied for one hour to rinsed sections and then washed for 2 minutes with SB-T at 1–5°C.

### Antibody staining method

5 µm frozen tissue sections were immediately placed onto a charged slide (Superfrost Plus), and the slides were then immersed in a 1–5°C 100% acetone fixative solution for at least 30 minutes. The slides were removed from the chilled acetone bath and immediately placed into 1–5°C, 1% paraformaldehyde/PBS pH 7.4 fixative solution for 8 minutes. After fixation the remaining OCT embedding medium was removed from slides by two 30-second rinses in tap water followed by a 5-minute rinse in selection buffer SB-T, pH 7.4. The tissue sections were then blocked for 30 minutes with 10% goat serum in SB-T, pH 7.4/50 mM glycine. Rabbit and mouse primary antibodies were diluted into SB-T 7.4 buffer and applied to the pre-blocked slides for at least one hour. Primary antibodies were washed from the slides for 5 minutes with SB-T, pH 7.4 and then incubated with either Dy549-goat anti-rabbit or Dy549-goat anti-mouse fluorescent secondary antibodies in SB-T, pH 7.4/300 nM DAPI for 30 minutes. The working concentration of the secondary antibody was 3 µg/ml. The slides were finally rinsed for 5 minutes in SB-T, pH 7.4 at room temperature and cover slipped with Fluoromount-G supplemented with 15 mM n-propyl gallate antifade reagent. Details regarding the antibodies used are given in Table 5.

**Table 5:** Antibodies used in for confirmation of analyte presence.

Antibody Target	Primary antibody dilution	Vendor	Location	Host	Poly/Mono-clonal
TSP-2	1∶1000 (0.35 µg/ml)	Novus Biologicals	Littleton, CO	rabbit	poly
TSP-2	1∶500	Anti-TSP-2 clone 67, a gift from Dr. Paul Bornstein	Seattle, WA	rabbit	poly
MMR/CD206	1∶500 (0.5 µg/ml)	R&D systems	Minneapolis, MN	rat	mono
ESAM	1∶250 (1 µg/ml)	R&D systems	Minneapolis, MN	mouse	mono

### Nikon 80i widefield microscope method

Fluorescence images were acquired with a Nikon 80i upright microscope equipped with Digital Sight DS-Ri1 color camera, mercury lamp, and optical filters appropriate for 4′,6-diamidino-2-phenylindole, dihydrochloride (DAPI) and Cy3 imaging. Data were collected and analyzed with Nikon NIS Elements AR 3.2 software.

### Nikon A1R confocal microscope method

Fluorescence images were acquired with a Nikon A1R ECLIPSE Ti-E inverted microscope equipped with a 32-channel PMT spectral detector unit (SDU). The Nikon SDU was used to subtract the signal arising from autofluorescence. The confocal imaging data was processed offline using the ROI spectral unmixing feature provided in the Nikon NIS Elements AR 3.2 software.

### Image processing method

ESAM expression was quantified and scored in single cells with CellProfiler and CellProfiler Analyst, respectively. The version number of CellProfiler software used for this analysis was r10997 and can be downloaded at www.cellprofiler.org [37]. Image analysis was performed using 10× images acquired on a Nikon 80i DS-Ri1 color camera at full frame 4076×3116 pixel dimensions. CellProfiler quantified the number of ESAM+ cells, defined as cells with DAPI+ nuclei and ESAM+ immunofluorescence, after a flat illumination field correction. The binary classification measurement rule was generated with a gentle boosting based supervised machine learning classifier module in CellProfiler Analysis r1123011246 [38].

## Supporting Information

Table S1 **SomaLogic Selection Targets.** The proteins against which each of the SOMAmers used in the SOMAscan assay were selected is listed.
(DOCX)
