# Supplementary material for: Protein Signature of Lung Cancer Tissues
Source: PLoS One. 2012 Apr 11;7(4):e35157. doi: 10.1371/journal.pone.0035157 (PMC3324437; doi:10.1371/journal.pone.0035157)
Supplement: Table S1 — SomaLogic Selection Targets. The proteins against which each of the SOMAmers used in the SOMAscan assay were selected is listed. (DOCX) [file pone.0035157.s001.docx]

| Table S1. SomaLogic Selection Targets | | |
| --- | --- | --- |
| Target | Uni-Prot Protein Name | Uni-Prot Acc # |
| 14-3-3 protein zeta/delta | 14-3-3 protein zeta/delta | P63104 |
| 3-phosphoinositide-dependent protein kinase 1 | 3-phosphoinositide-dependent protein kinase 1 | O15530 |
| 4-1BB ligand/CD137L | Tumor necrosis factor ligand superfamily member 9 | P41273 |
| 4-1BB/CD137 | Tumor necrosis factor receptor superfamily member 9 | Q07011 |
| 6Ckine/CCL21 | Small-inducible cytokine A21 | O00585 |
| α1-Antichymotrypsin | Alpha-1-antichymotrypsin | P01011 |
| α1-Antitrypsin | Alpha-1-antitrypsin | P01009 |
| α2-Antiplasmin | Alpha-2-antiplasmin | P08697 |
| α2-HS-Glycoprotein | Alpha-2-HS-glycoprotein | P02765 |
| α2-Macroglobulin | Alpha-2-macroglobulin | P01023 |
| α-2-macroglobulin receptor-associated protein | Alpha-2-macroglobulin receptor-associated protein | P30533 |
| α-L-iduronidase | Alpha-L-iduronidase | P35475 |
| Acid ceramidase-like protein | N-acylethanolamine-hydrolyzing acid amidase | Q02083 |
| Acid phosphatase 1, soluble | Low molecular weight phosphotyrosine protein phosphatase | P24666 |
| Acidic fibroblast growth factor/β-endothelial cell growth factor | Heparin-binding growth factor 1 | P05230 |
| Activated leukocyte cell adhesion molecule | Activated leukocyte cell adhesion molecule | Q13740 |
| Activated Protein C | Vitamin K-dependent protein C (activated form) | P04070 |
| Activin A/Inhibin β-A homodimer | Inhibin beta A chain | P08476 |
| Activin receptor-like kinase 1 | Serine/threonine-protein kinase receptor R3 | P37023 |
| Activin Serine-threonine-protein kinase receptor type-1B | Activin receptor type-1B | P36896 |
| ADAM metallopeptidase domain 9 | Disintegrin and metalloproteinase domain-containing protein 9 | Q13443 |
| ADAM metallopeptidase with thrombospondin motifs 1 | ADAMTS-1 | Q9UHI8 |
| ADAM metallopeptidase with thrombospondin motifs 13 | ADAMTS-13 | Q76LX8 |
| ADAM metallopeptidase with thrombospondin motifs 4/Aggrecanase 1 | ADAMTS-4 | O75173 |
| ADAM metallopeptidase with thrombospondin motifs 5/Aggrecanase 2 | ADAMTS-5 | Q9UNA0 |
| Adenosylhomocysteinase | Adenosylhomocysteinase | P23526 |
| Adiponectin | Adiponectin | Q15848 |
| Adrenocorticotropic hormone | Beta-endorphin | P01189 |
| AGGF1 | Angiogenic factor with G patch and FHA domains 1 | Q8N302 |
| Aggrecan core protein | Aggrecan core protein | P16112 |
| Agouti-related protein | Agouti-related protein | O00253 |
| AH receptor-interacting protein | AH receptor-interacting protein | O00170 |
| Alanine aminotransaminase 1 | Alanine aminotransferase 1 | P24298 |
| Albumin | Serum albumin | P02768 |
| Alkaline phosphatase, tissue-nonspecific isozyme | Alkaline phosphatase, tissue-nonspecific isozyme | P05186 |
| Allograft inflammatory factor 1 | Allograft inflammatory factor 1 | P55008 |
| Aminoacylase-1 | Aminoacylase-1 | Q03154 |
| Amphiregulin | Amphiregulin | P15514 |
| Amyloid β A4 protein | Amyloid beta A4 protein | P05067 |
| Angiogenin | Angiogenin | P03950 |
| Angiopoietin-1 | Angiopoietin-1 | Q15389 |
| Angiopoietin-2 | Angiopoietin-2 | O15123 |
| Angiopoietin-4 | Angiopoietin-4 | Q9Y264 |
| Angiopoietin-related 3 | Angiopoietin-related protein 3 | Q9Y5C1 |
| Angiopoietin-related 4 | Angiopoietin-related protein 4 | Q9BY76 |
| Angiostatin | Angiostatin | P00747 |
| Angiotensin-converting enzyme 2 | Angiotensin-converting enzyme 2 | Q9BYF1 |
| Angiotensinogen | Angiotensinogen | P01019 |
| Antithrombin III | Antithrombin-III | P01008 |
| Apolipoprotein A-I | Apolipoprotein A-I | P02647 |
| Apolipoprotein B | Apolipoprotein B-100 and Apolipoprotein B-48 | P04114 |
| Apolipoprotein E | Apolipoprotein E | P02649 |
| Apolipoprotein E receptor 2/LRP8 | Low-density lipoprotein receptor-related protein 8 | Q14114 |
| Apolipoprotein E2 | Apolipoprotein E (isoform E2) | P02649 |
| Apolipoprotein E3 | Apolipoprotein E (isoform E3) | P02649 |
| Apolipoprotein E4 | Apolipoprotein E (isoform E4) | P02649 |
| Apoptosis regulator Bcl-2 | Apoptosis regulator Bcl-2 | P10415 |
| APRIL/A proliferation inducing ligand | Tumor necrosis factor ligand superfamily member 13 | O75888 |
| Arginase-1 | Arginase-1 | P05089 |
| ARID domain-containing protein 3A | AT-rich interactive domain-containing protein 3A | Q99856 |
| Artemin | Artemin | Q5T4W7 |
| Arylsulfatase A | Arylsulfatase A | P15289 |
| Arylsulfatase B | Arylsulfatase B | P15848 |
| Asialoglycoprotein receptor 1 | Asialoglycoprotein receptor 1 | P07306 |
| ATP-dependent DNA helicase II 70 kDa subunit | ATP-dependent DNA helicase 2 subunit 1 | P12956 |
| Aurora kinase A | Serine/threonine-protein kinase 6 | O14965 |
| Aurora-related kinase 2 | Serine/threonine-protein kinase 12 | Q96GD4 |
| Azurocidin | Azurocidin | P20160 |
| β2-Microglobulin | Beta-2-microglobulin | P61769 |
| β-adrenergic receptor kinase 1 | Beta-adrenergic receptor kinase 1 | P25098 |
| β-nerve growth factor | Beta-nerve growth factor | P01138 |
| B lymphocyte chemoattractant/CXCL13/BCA-1 | Small-inducible cytokine B13 | O43927 |
| Bactericidal permeability-increasing protein | Bactericidal permeability-increasing protein | P17213 |
| Basal Cell Adhesion Molecule | Lutheran blood group glycoprotein | P50895 |
| Basic fibroblast growth factor | Heparin-binding growth factor 2 | P09038 |
| Basic fibroblast growth factor receptor 1 | Basic fibroblast growth factor receptor 1 | P11362 |
| B-cell maturation protein | Tumor necrosis factor receptor superfamily member 17 | Q02223 |
| B-cell-activating factor | Tumor necrosis factor ligand superfamily member 13B | Q9Y275 |
| B-cell-activating factor receptor | Tumor necrosis factor receptor superfamily member 13C | Q96RJ3 |
| Bcl-2-related protein A1 | Bcl-2-related protein A1 | Q16548 |
| Biglycan | Biglycan | P21810 |
| Bone morphogenetic protein receptor type IA | Bone morphogenetic protein receptor type IA | P36894 |
| Bone morphogenetic protein type II receptor | Bone morphogenetic protein receptor type-2 | Q13873 |
| Bone morphogenetic protein-1 | Bone morphogenetic protein 1 | P13497 |
| Bone morphogenetic protein-6 | Bone morphogenetic protein 6 | P22004 |
| Bone morphogenetic protein-7 | Bone morphogenetic protein 7 | P18075 |
| Bone morphogenetic protein-10 | Bone morphogenetic protein 10 | O95393 |
| Bone morphogenetic protein-14 | Growth/differentiation factor 5 | P43026 |
| Bone morphogenetic protein-binding endothelial regulator protein | BMP-binding endothelial regulator protein | Q8N8U9 |
| Bone proteoglycan II | Decorin | P07585 |
| Bone sialoprotein 2 | Bone sialoprotein 2 | P21815 |
| Brain natriuretic peptide 32 | Brain natriuretic peptide 32 | P16860 |
| Brain-derived neurotrophic factor | Brain-derived neurotrophic factor | P23560 |
| Brevican | Brevican core protein | Q96GW7 |
| c-abl oncogene 1, non-receptor tyrosine kinase | Proto-oncogene tyrosine-protein kinase ABL1 | P00519 |
| Cadherin-1 | Epithelial cadherin | P12830 |
| Cadherin-2 | Neural cadherin | P19022 |
| Cadherin-3 | Cadherin-3 | P22223 |
| Cadherin-5 | Vascular endothelial cadherin | P33151 |
| Cadherin-6 | Kidney cadherin | P55285 |
| Cadherin-12 | Brain cadherin | P55289 |
| Calcineurin B α | Calcineurin subunit B type 1 | P63098 |
| Calcium-calmodulin-dependent protein kinase I | Calcium/calmodulin-dependent protein kinase type 1 | Q14012 |
| Calcium-calmodulin-dependent protein kinase ID | Calcium/calmodulin-dependent protein kinase type 1D | Q8IU85 |
| Calcium-calmodulin-dependent protein kinase II α | Calcium/calmodulin-dependent protein kinase type II alpha chain | Q9UQM7 |
| Calcium-calmodulin-dependent protein kinase II β | Calcium/calmodulin-dependent protein kinase type II beta chain | Q13554 |
| Calcium-calmodulin-dependent protein kinase II δ | Calcium/calmodulin-dependent protein kinase type II delta chain | Q13557 |
| Calcium-calmodulin-dependent protein kinase kinase 1, α | Calcium/calmodulin-dependent protein kinase kinase 1 | Q8N5S9 |
| Calpain I | Calpain-1 catalytic subunit and Calpain small subunit 1 | P07384, P04632 |
| Calpastatin | Calpastatin | P20810 |
| cAMP-dependent protein kinase catalytic subunit α | cAMP-dependent protein kinase catalytic subunit alpha | P17612 |
| Carbonic anhydrase III | Carbonic anhydrase 3 | P07451 |
| Carbonic anhydrase IV | Carbonic anhydrase 4 | P22748 |
| Carbonic anhydrase IX | Carbonic anhydrase 9 | Q16790 |
| Carbonic anhydrase VI | Carbonic anhydrase 6 | P23280 |
| Carbonic anhydrase VII | Carbonic anhydrase 7 | P43166 |
| Carbonic anhydrase XIII | Carbonic anhydrase 13 | Q8N1Q1 |
| Carbonic anhydrase-related protein X | Carbonic anhydrase-related protein 10 | Q9NS85 |
| Cardiotrophin-1 | Cardiotrophin-1 | Q16619 |
| Carnosine dipeptidase 1 | Beta-Ala-His dipeptidase | Q96KN2 |
| Casein kinase II subunit α | Casein kinase II subunit alpha | P68400 |
| Caspase-3 | Caspase-3 (pro form) | P42574 |
| Catalase | Catalase | P04040 |
| Cathepsin A | Lysosomal protective protein | P10619 |
| Cathepsin B | Cathepsin B | P07858 |
| Cathepsin C | Dipeptidyl-peptidase 1 | P53634 |
| Cathepsin D | Cathepsin D | P07339 |
| Cathepsin E | Cathepsin E | P14091 |
| Cathepsin G | Cathepsin G | P08311 |
| Cathepsin H | Cathepsin H | P09668 |
| Cathepsin S | Cathepsin S | P25774 |
| Cathepsin V | Cathepsin L2 | O60911 |
| CC chemokine I-309/CCL1 | Small-inducible cytokine A1 | P22362 |
| CCL28 | Small-inducible cytokine A28 | Q9NRJ3 |
| CD22 | B-cell receptor CD22 | P20273 |
| CD23 | Low affinity immunoglobulin epsilon Fc receptor | P06734 |
| CD30 | Tumor necrosis factor receptor superfamily member 8 | P28908 |
| CD30 Ligand | Tumor necrosis factor ligand superfamily member 8 | P32971 |
| CD36 ANTIGEN | Platelet glycoprotein 4 | P16671 |
| CD39 | Ectonucleoside triphosphate diphosphohydrolase 1 | P49961 |
| CD40 ligand | CD40 ligand | P29965 |
| CD48 | CD48 antigen | P09326 |
| CD5 antigen-like | CD5 antigen-like | O43866 |
| CD70 | CD70 antigen | P32970 |
| CD97 | CD97 antigen | P48960 |
| CD109 | CD109 antigen | Q6YHK3 |
| CEA | Carcinoembryonic antigen-related cell adhesion molecule 5 | P06731 |
| Chemerin | Retinoic acid receptor responder protein 2 | Q99969 |
| Chitotriosidase-1 | Chitotriosidase-1 | Q13231 |
| Chordin-Like 1 | Chordin-like protein 1 | Q9BU40 |
| Chymase | Chymase | P23946 |
| Ciliary Neurotrophic Factor | Ciliary neurotrophic factor | P26441 |
| Ciliary neurotrophic factor receptor α | Ciliary neurotrophic factor receptor alpha | P26992 |
| Ck-β-8-1/Macrophage inflammatory protein 3 splice variant (aa 46-137) | Small-inducible cytokine A23 | P55773 |
| CN166 | UPF0568 protein C14orf166 | Q9Y224 |
| Coagulation Factor V | Coagulation factor V | P12259 |
| Coagulation Factor VII | Coagulation factor VII | P08709 |
| Coagulation Factor IX | Coagulation factor IX | P00740 |
| Coagulation Factor IXab | Coagulation factor IX (activated form) | P00740 |
| Coagulation Factor X | Coagulation factor X | P00742 |
| Coagulation Factor Xa | Coagulation factor X (activated form) | P00742 |
| Coagulation Factor XI | Coagulation factor XI | P03951 |
| Coiled-coil domain-containing protein 80/URB | Coiled-coil domain-containing protein 80 | Q76M96 |
| Collectin placenta 1 | Collectin-12 | Q5KU26 |
| COMM domain containing 7 | COMM domain-containing protein 7 | Q86VX2 |
| Complement C1q | Complement C1q subcomponent subunits A, B, and C | P02747, P02746, P02745 |
| Complement C1r | Complement C1r subcomponent | P00736 |
| Complement C1s | Complement C1s subcomponent | P09871 |
| Complement C2 | Complement C2 | P06681 |
| Complement C3 | Complement C3 | P01024 |
| Complement C3a anaphylatoxin | C3a anaphylatoxin | P01024 |
| Complement C3a anaphylatoxin des Arginine | C3a anaphylatoxin des Arginine | P01024 |
| Complement C3b | Complement C3b | P01024 |
| Complement C3b, inactivated | Complement C3b, incactivated | P01024 |
| Complement C3d | Complement C3d fragment | P01024 |
| Complement C4 | Complement C4-A and Complement C4-B | P0C0L4, P0C0L5 |
| Complement C4b | C4b-A | P0C0L4 P0C0L5 |
| Complement C5 | Complement C5 | P01031 |
| Complement C5a | C5a anaphylatoxin | P01031 |
| Complement C5b,6 Complex | Complement C5b, and Complement component C6 | P01031 P13671 |
| Complement C6 | Complement component C6 | P13671 |
| Complement C7 | Complement component C7 | P10643 |
| Complement C8 | Complement component C8 alpha, beta, and gamma chains | P07357, P07358, P07360 |
| Complement C9 | Complement component C9 | P02748 |
| Complement factor B | Complement factor B | P00751 |
| Complement factor D | Complement factor D | P00746 |
| Complement factor H | Complement factor H | P08603 |
| Complement factor H-related 5 | Complement factor H-related protein 5 | Q9BXR6 |
| Complement factor I | Complement factor I | P05156 |
| Connective tissue growth factor | Connective tissue growth factor | P29279 |
| Contactin-1 | Contactin-1 | Q12860 |
| Contactin-2 | Contactin-2 | Q02246 |
| Contactin-4 | Contactin-4 | Q8IWV2 |
| Contactin-5 | Contactin-5 | O94779 |
| C-reactive protein | C-reactive protein | P02741 |
| Creatine kinase-BB | Creatine kinase B-type | P12277 |
| Creatine kinase-MB | Creatine kinase B-type, Creatine kinase M-type | P12277, P06732 |
| Creatine kinase-MM | Creatine kinase M-type | P06732 |
| Cripto-1 | Teratocarcinoma-derived growth factor 1 | P13385 |
| Cryptic protein | Cryptic protein | P0CG37 |
| C-Src kinase | Tyrosine-protein kinase CSK | P41240 |
| Cutaneous T-cell-attracting chemokine/CCL27 | Small-inducible cytokine A27 | Q9Y4X3 |
| Cyclin-dependent kinase 1:cyclin B complex | Cell division control protein 2 homolog, G2/mitotic-specific cyclin-B1 Complex | P06493, P14635 |
| Cyclin-dependent kinase 2:cyclin A complex | Cell division protein kinase 2, Cyclin-A2 Complex | P24941, P20248 |
| Cyclin-dependent kinase 5:activator p35 complex | Cell division protein kinase 5, Cyclin-dependent kinase 5 activator 1, p35 Complex | Q00535, Q15078 |
| Cyclin-dependent kinase 8:cyclin C complex | Cell division protein kinase 8, Cyclin-C Complex | P49336, P24863 |
| Cyclin-dependent kinase inhibitor p27 | Cyclin-dependent kinase inhibitor 1B | P46527 |
| Cyclooxygenase-2 | Prostaglandin G/H synthase 2 | P35354 |
| Cyclophilin A | Peptidyl-prolyl cis-trans isomerase A | P62937 |
| Cystatin C | Cystatin-C | P01034 |
| Cystatin D | Cystatin-D | P28325 |
| Cystatin F | Cystatin-F | O76096 |
| Cystatin M | Cystatin-M | Q15828 |
| Cystatin S | Cystatin-S | P01036 |
| Cystatin SN | Cystatin-SN | P01037 |
| Cysteine-rich secretory protein 3 | Cysteine-rich secretory protein 3 | P54108 |
| Cytochrome c | Cytochrome c | P99999 |
| Cytochrome P450 3A4 | Cytochrome P450 3A4 | P08684 |
| Cytokine receptor-like factor 1:Cardiotrophin-like cytokine factor 1 Complex | Cytokine receptor-like factor 1 and Cardiotrophin-like cytokine factor 1 | O75462, Q9UBD9 |
| Cytotoxic T-lymphocyte-4 | Cytotoxic T-lymphocyte protein 4 | P16410 |
| DARPP-32 | Protein phosphatase 1 regulatory subunit 1B | Q9UD71 |
| DEAD box RNA helicase 19B | ATP-dependent RNA helicase DDX19B | Q9UMR2 |
| Death-associated protein kinase 2 | Death-associated protein kinase 2 | Q9UIK4 |
| Dectin-1 | C-type lectin domain family 7 member A | Q9BXN2 |
| Dendritic cell-specific ICAM-3-grabbing nonintegrin 1/CD209 | CD209 antigen | Q9NNX6 |
| Dendritic cell-specific ICAM-3-grabbing nonintegrin 2/CD299 | C-type lectin domain family 4 member M | Q9H2X3 |
| Dentin matrix protein 1 | Dentin matrix acidic phosphoprotein 1 | Q13316 |
| Desert Hedgehog N-Terminus | Desert hedgehog protein precursor | O43323 |
| Desmoglein-1 | Desmoglein-1 | Q02413 |
| Diablo, IAP-binding mitochondrial protein | Diablo homolog, mitochondrial | Q9NR28 |
| Dickkopf-related protein 1 | Dickkopf-related protein 1 | O94907 |
| Dickkopf-related protein 3 | Dickkopf-related protein 3 | Q9UBP4 |
| Dickkopf-related protein 4 | Dickkopf-related protein 4 | Q9UBT3 |
| Dipeptidyl-peptidase II | Dipeptidyl-peptidase 2 | Q9UHL4 |
| Discoidin domain receptor 1 | Epithelial discoidin domain-containing receptor 1 | Q08345 |
| Discoidin domain receptor 2 | Discoidin domain-containing receptor 2 | Q16832 |
| DNA repair protein RAD51 homolog 1 | DNA repair protein RAD51 homolog 1 | Q06609 |
| Dopa decarboxylase | Aromatic-L-amino-acid decarboxylase | P20711 |
| Dopamine responsive protein | Vacuolar protein sorting-associated protein VTA1 homolog | Q9NP79 |
| Down-regulated in renal cell carcinoma 1 | Protein FAM107A | O95990 |
| Drosophila Delta homolog 4 | Delta-like protein 4 | Q9NR61 |
| Dual-specificity protein kinase 3 | Dual specificity tyrosine-phosphorylation-regulated kinase 3 | O43781 |
| Dynein light chain 1 | Dynein light chain 1, cytoplasmic | P63167 |
| Dynein light chain roadblock-type 1 | Dynein light chain roadblock-type 1 | Q9NP97 |
| Ectodermal Dysplasia Receptor | Tumor necrosis factor receptor superfamily member EDAR | Q9UNE0 |
| Ectodysplasin-A, secreted form | Ectodysplasin-A (splice variant A2) | Q92838 |
| Elastase | Leukocyte elastase | P08246 |
| Elongation factor 1-β | Elongation factor 1-beta | P24534 |
| Elongation factor 1-γ | Elongation factor 1-gamma | P26641 |
| Endocan | cDNA FLJ50870, moderately similar to Endothelial cell-specific molecule 1 | Q9NQ30 |
| Endocrine-gland-derived vascular endothelial growth factor | Prokineticin-1 | P58294 |
| Endostatin | Endostatin | P39060 |
| Endothelial cell-selective adhesion molecule/ESAM | Endothelial cell-selective adhesion molecule | Q96AP7 |
| Endothelial-Monocyte Activating Polypeptide 2 | Endothelial monocyte-activating polypeptide 2 | Q12904 |
| Endothelin-converting enzyme 1 | Endothelin-converting enzyme 1 | P42892 |
| Enterokinase | Enteropeptidase | P98073 |
| Eotaxin | Eotaxin | P51671 |
| Eotaxin-2 | Small-inducible cytokine A24 | O00175 |
| Ephrin type-A receptor 1 | Ephrin type-A receptor 1 | P21709 |
| Ephrin type-A receptor 2 | Ephrin type-A receptor 2 | P29317 |
| Ephrin type-A receptor 3 | Ephrin type-A receptor 3 | P29320 |
| Ephrin type-A receptor 5 | Ephrin type-A receptor 5 | P54756 |
| Ephrin type-B receptor 4 | Ephrin type-B receptor 4 | P54760 |
| Ephrin-A4 | Ephrin-A4 | P52798 |
| Ephrin-A5 | Ephrin-A5 | P52803 |
| Ephrin-B3 | Ephrin-B3 | Q15768 |
| Epithelial-derived neutrophil-activating protein 78/CXCL5 | C-X-C motif chemokine 5 | P42830 |
| erbB1/HER1 | Epidermal growth factor receptor | P00533 |
| erbB2 /HER2 | Receptor tyrosine-protein kinase erbB-2 | P04626 |
| erbB3/HER3 | Receptor tyrosine-protein kinase erbB-3 | P21860 |
| erbB4/HER4 | Receptor tyrosine-protein kinase erbB-4 | Q15303 |
| Erythropoietin | Erythropoietin | P01588 |
| Erythropoietin receptor | Erythropoietin receptor | P19235 |
| E-Selectin | E-selectin | P16581 |
| Estrogen receptor | Estrogen receptor | P03372 |
| Ethylmalonic encephalopathy 1 | Protein ETHE1, mitochondrial | O95571 |
| Eukaryotic translation initiation factor 4H | Eukaryotic translation initiation factor 4H | Q15056 |
| Eukaryotic translation initiation factor 5 | Eukaryotic translation initiation factor 5 | P55010 |
| Eukaryotic translation initiation factor 5A | Eukaryotic translation initiation factor 5A-1 | P63241 |
| Extracellular matrix metalloproteinase inducer | Basigin | P35613 |
| Extracellular matrix protein-1 | Extracellular matrix protein 1 | Q16610 |
| Fas ligand | Tumor necrosis factor ligand superfamily member 6 | P48023 |
| Ferritin | Ferritin heavy and light chains | P02794, P02792 |
| Fetuin B | Fetuin-B | Q9UGM5 |
| Fibrinogen | Fibrinogen alpha, beta, and gamma chains | P02671, P02675, P02679 |
| Fibroblast growth factor 4 | Fibroblast growth factor 4 | P08620 |
| Fibroblast growth factor 5 | Fibroblast growth factor 5 | P12034 |
| Fibroblast growth factor 6 | Fibroblast growth factor 6 | P10767 |
| Fibroblast growth factor 7 | Keratinocyte growth factor | P21781 |
| Fibroblast growth factor 8 isoform A | Fibroblast growth factor 8 - isoform 8B | P55075 |
| Fibroblast growth factor 9 | Glia-activating factor | P31371 |
| Fibroblast growth factor 10/Keratinocyte growth factor 2 | Fibroblast growth factor 10 | O15520 |
| Fibroblast growth factor 16 | Fibroblast growth factor 16 | O43320 |
| Fibroblast growth factor 17 | Fibroblast growth factor 17 | O60258 |
| Fibroblast growth factor 18 | Fibroblast growth factor 18 | O76093 |
| Fibroblast growth factor 19 | Fibroblast growth factor 19 | O95750 |
| Fibroblast growth factor 20 | Fibroblast growth factor 20 | Q9NP95 |
| Fibroblast growth factor 23 | Fibroblast growth factor 23 | Q9GZV9 |
| Fibroblast growth factor receptor 2 | Fibroblast growth factor receptor 2 | P21802 |
| Fibroblast growth factor receptor 3 | Fibroblast growth factor receptor 3 | P22607 |
| Fibronectin | Fibronectin | P02751 |
| Fibronectin-1 Fragment 3 | Fibronectin-1 Fragment 3 | P02751 |
| Fibronectin-1 Fragment 4 | Fibronectin-1 Fragment 4 | P02751 |
| Ficolin-1 | Ficolin-1 | O00602 |
| Ficolin-2 | Ficolin-2 | Q15485 |
| Ficolin-3 | Ficolin-3 | O75636 |
| Fms-related tyrosine kinase 3 ligand | SL cytokine | P49771 |
| Follicle stimulating hormone | Glycoprotein hormones alpha chain and Follitropin subunit beta | P01215, P01225 |
| Follistatin | Follistatin | P19883 |
| Follistatin-like 3 | Follistatin-related protein 3 | O95633 |
| Fortilin | Translationally-controlled tumor protein | P13693 |
| Fractalkine/CX3CL-1 | Fractalkine | P78423 |
| Frizzled-related protein 1, secreted | Secreted frizzled-related protein 1 | Q8N474 |
| Frizzled-related protein 3, secreted | Secreted frizzled-related protein 3 | Q92765 |
| Fructose-bisphosphate aldolase A | Fructose-bisphosphate aldolase A | P04075 |
| G protein-coupled receptor associated sorting protein 2 | G-protein coupled receptor-associated sorting protein 2 | Q96D09 |
| Galectin-2 | Galectin-2 | P05162 |
| Galectin-3 | Galectin-3 | P17931 |
| Galectin-4 | Galectin-4 | P56470 |
| Gastrin-releasing peptide | Gastrin-releasing peptide | P07492 |
| GDNF family receptor α1 | GDNF family receptor alpha-1 | P56159 |
| GDNF family receptor α2 | GDNF family receptor alpha-2 | O00451 |
| GDNF family receptor α3 | GDNF family receptor alpha-3 | O60609 |
| Glial cell line-derived neurotrophic factor | Glial cell line-derived neurotrophic factor | P39905 |
| Glial fibrillary acidic protein | Glial fibrillary acidic protein | P14136 |
| Glucocorticoid receptor | Glucocorticoid receptor | P04150 |
| Glutamate carboxypeptidase | Cytosolic non-specific dipeptidase | Q96KP4 |
| Glyceraldehyde-3-phosphate dehydrogenase | Glyceraldehyde-3-phosphate dehydrogenase | P04406 |
| Glycogen synthase kinase-3 α | Glycogen synthase kinase-3 alpha | P49840 |
| Glycogen synthase kinase-3 β | Glycogen synthase kinase-3 beta | P49841 |
| Glypican-2 | Glypican-2 | Q8N158 |
| Glypican 3 | Glypican-3 | P51654 |
| GPVI/Platelet Glycoprotein VI | Platelet glycoprotein VI | Q9HCN6 |
| Granulocyte chemotactic protein 2/CXCL6 | C-X-C motif chemokine 6 | P80162 |
| Granulocyte colony-stimulating factor receptor | Granulocyte colony-stimulating factor receptor | Q99062 |
| Granulysin | Granulysin | P22749 |
| Granzyme A | Granzyme A | P12544 |
| Granzyme B | Granzyme B | P10144 |
| Granzyme H | Granzyme H | P20718 |
| Growth and differentiation factor-associated serum protein 1 | WAP, kazal, immunoglobulin, kunitz and NTR domain-containing protein 2 | Q8TEU8 |
| Growth Arrest Specific 1 | Growth arrest-specific protein 1 | P54826 |
| Growth hormone receptor | Growth hormone receptor | P10912 |
| Growth-differentiation factor 9 | Growth/differentiation factor 9 | O60383 |
| Growth-differentiation factor 11 | Growth/differentiation factor 11 | O95390 |
| Gro-α | Growth-regulated alpha protein | P09341 |
| Gro-β | Macrophage inflammatory protein 2-alpha | P19875 |
| Gro-γ | Macrophage inflammatory protein 2-beta | P19876 |
| Haptoglobin | Haptoglobin | P00738 |
| HE4 | WAP four-disulfide core domain protein 2 | Q14508 |
| Heme oxygenase 2 | Heme oxygenase 2 | P30519 |
| Hemofiltrate CC Chemokine 1/CCL14 | C-C motif chemokine 14 | Q16627 |
| Hemojuvelin | Hemojuvelin | Q6ZVN8 |
| Hemopexin | Hemopexin | P02790 |
| Hemopoietic cell kinase | Tyrosine-protein kinase HCK | P08631 |
| Heparin cofactor II | Heparin cofactor 2 | P05546 |
| Heparin-binding EGF-like growth factor | Heparin-binding EGF-like growth factor | Q99075 |
| Hepatocyte growth factor | Hepatocyte growth factor | P14210 |
| Hepatocyte growth factor activator | Hepatocyte growth factor activator | Q04756 |
| Hepatocyte growth factor activator inhibitor type 1 | Kunitz-type protease inhibitor 1 | O43278 |
| Hepatocyte growth factor receptor/c-Met | Hepatocyte growth factor receptor | P08581 |
| High affinity Immunoglobulin G Fc receptor I | High affinity immunoglobulin gamma Fc receptor I | P12314 |
| High temperature requirement serine peptidase A2 | Serine protease HTRA2, mitochondrial | O43464 |
| High-mobility group box 1/amphoterin | High mobility group protein B1 | P09429 |
| Histidine triad nucleotide binding protein 1 | Histidine triad nucleotide-binding protein 1 | P49773 |
| Histone acetyltransferase 1 | Histone acetyltransferase type B catalytic subunit | O14929 |
| Histone acetyltransferases monocytic leukemic zinc-finger protein | Histone acetyltransferase MYST3 | Q92794 |
| Histone deacetylase 8 | Histone deacetylase 8 | Q9BY41 |
| Histone H1.2 | Histone H1.2 | P16403 |
| Histone H2A.z | Histone H2A.Z | P0C0S5 |
| Histone H3-K9 methyltransferase 3 | Histone-lysine N-methyltransferase, H3 lysine-9 specific 3 | Q96KQ7 |
| HIV-2 Rev | Protein Rev (Human Immunodeficiency Virus) | P18093 |
| Homeobox transcription factor Nanog | Homeobox protein NANOG | Q9H9S0 |
| Homeodomain-interacting protein kinase 3 | Homeodomain-interacting protein kinase 3 | Q9H422 |
| Hsp40 | DnaJ homolog subfamily B member 1 | P25685 |
| Hsp60 | 60 kDa heat shock protein, mitochondrial | P10809 |
| Hsp70 | Heat shock 70 kDa protein 1 | P08107 |
| Hsp90 co-chaperone Cdc37 | Hsp90 co-chaperone Cdc37 | Q16543 |
| Hsp90α | Heat shock protein HSP 90-alpha | P07900 |
| Hsp90β | Heat shock protein HSP 90-beta | P08238 |
| Hyaluronan and proteoglycan link protein 1 | Hyaluronan and proteoglycan link protein 1 | P10915 |
| Iduronate 2-sulfatase | Iduronate 2-sulfatase | P22304 |
| Immunoglobulin E | Immunoglobulin E | P01854 |
| Immunoglobulin G | Immunoglobulin G | P01857 |
| Immunoglobulin G Fc region receptor II-a, low affinity | Low affinity immunoglobulin gamma Fc region receptor II-a | P12318 |
| Immunoglobulin G Fc region receptor II-b, ow affinity | Low affinity immunoglobulin gamma Fc region receptor II-b | P31994 |
| Immunoglobulin G Fc region receptor III-B, low affinity | Low affinity immunoglobulin gamma Fc region receptor III-B | O75015 |
| Immunoglobulin M | Immunoglobulin M | P01871 |
| Importin β1 | Importin subunit beta-1 | Q14974 |
| Inducible T-cell co-stimulator | Inducible T-cell co-stimulator | Q9Y6W8 |
| Inferferon-α2 | Interferon alpha-2 | P01563 |
| Inferferon-γ | Interferon gamma | P01579 |
| Inferferon-γ Receptor 1 | Interferon-gamma receptor alpha chain | P15260 |
| Inferferon-λ 1 | Interleukin-29 | Q8IU54 |
| Inferferon-λ 2 | Interleukin-28A | Q8IZJ0 |
| Inhibitor of growth 1 | Inhibitor of growth protein 1 | Q9UK53 |
| Insulin receptor | Insulin receptor | P06213 |
| Insulin-degrading enzyme/Insulysin | Insulin-degrading enzyme | P14735 |
| Insulin-like growth factor I | Insulin-like growth factor IA and IB | P05019 |
| Insulin-like growth factor II receptor | Cation-independent mannose-6-phosphate receptor | P11717 |
| Insulin-like growth factor-binding protein 1 | Insulin-like growth factor-binding protein 1 | P08833 |
| Insulin-like growth factor-binding protein 2/IGFBP-2 | Insulin-like growth factor-binding protein 2 | P18065 |
| Insulin-like growth factor-binding protein 3 | Insulin-like growth factor-binding protein 3 | P17936 |
| Insulin-like growth factor-binding protein 4 | Insulin-like growth factor-binding protein 4 | P22692 |
| Insulin-like growth factor-binding protein 5/IGFBP-5 | Insulin-like growth factor-binding protein 5 | P24593 |
| Insulin-like growth factor-binding protein 6 | Insulin-like growth factor-binding protein 6 | P24592 |
| Insulin-like growth factor-binding protein 7/IGFBP-7 | Insulin-like growth factor-binding protein 7 | Q16270 |
| Integrin a-IIb: ß-3 complex | Integrin alpha-Iib and Integrin beta-3 | P08514, P05106 |
| Integrin α-I: β-1 complex | Integrin alpha-1 and Integrin beta-1 | P56199, P05556 |
| Intercellular adhesion molecule 1 | Intercellular adhesion molecule 1 | P05362 |
| Intercellular adhesion molecule 2 | Intercellular adhesion molecule 2 | P13598 |
| Intercellular adhesion molecule 3 | Intercellular adhesion molecule 3 | P32942 |
| Interferon-γ induced protein | Small-inducible cytokine B10 | P02778 |
| Interferon-γ-inducible protein-9 | Small-inducible cytokine B11 | O14625 |
| Interleukin-1 receptor 1 | Interleukin-1 receptor type I | P14778 |
| Interleukin-1 receptor 4 | Interleukin-1 receptor-like 1 | Q01638 |
| Interleukin-1 Receptor accessory protein | Interleukin-1 receptor accessory protein | Q9NPH3 |
| Interleukin-1 receptor-like 2 | Interleukin-1 receptor-like 2 | Q9HB29 |
| Interleukin-1β | Interleukin-1 beta | P01584 |
| Interleukin-2 | Interleukin-2 | P60568 |
| Interleukin-2 receptor α chain | Interleukin-2 receptor alpha chain | P01589 |
| Interleukin-2 receptor γ chain | Cytokine receptor common gamma chain | P31785 |
| Interleukin-3 receptor α | Interleukin-3 receptor alpha chain | P26951 |
| Interleukin-4 | Interleukin-4 | P05112 |
| Interleukin-4 receptor α chain | Interleukin-4 receptor alpha chain | P24394 |
| Interleukin-5 | Interleukin-5 | P05113 |
| Interleukin-5 receptor α | Interleukin-5 receptor alpha chain | Q01344 |
| Interleukin-6 | Interleukin-6 | P05231 |
| Interleukin-6 receptor subunit β/gp130 | Interleukin-6 receptor subunit beta | P40189 |
| Interleukin-6 receptor α chain | Interleukin-6 receptor alpha chain | P08887 |
| Interleukin-7 | Interleukin-7 | P13232 |
| Interleukin-7 receptor α | Interleukin-7 receptor alpha chain | P16871 |
| Interleukin-8/IL-8 | Interleukin-8 | P10145 |
| Interleukin-9 | Interleukin-9 | P15248 |
| Interleukin-10 | Interleukin-10 | P22301 |
| Interleukin-10 receptor β | Interleukin-10 receptor beta chain | Q08334 |
| Interleukin-11 | Interleukin-11 | P20809 |
| Interleukin-11 receptor α | Interleukin-11 receptor alpha chain | Q14626 |
| Interleukin-12 | Interleukin-12 subunits alpha and beta | P29459, P29460 |
| Interleukin-12 receptor β1 | Interleukin-12 receptor beta-1 chain | P42701 |
| Interleukin-12 receptor β2 | Interleukin-12 receptor beta-2 chain | Q99665 |
| Interleukin-13 | Interleukin-13 | P35225 |
| Interleukin-13 receptor α1 | Interleukin-13 receptor alpha-1 chain | P78552 |
| Interleukin-15 receptor α | Interleukin-15 receptor alpha chain | Q13261 |
| Interleukin-16 | Interleukin-16 | Q14005 |
| Interleukin-17 | Interleukin-17A | Q16552 |
| Interleukin-17 receptor A | Interleukin-17 receptor A | Q96F46 |
| Interleukin-17 receptor C | Interleukin-17 receptor C | Q8NAC3 |
| Interleukin-17 receptor D | Interleukin-17 receptor D | Q8NFM7 |
| Interleukin-17B | Interleukin-17B | Q9UHF5 |
| Interleukin-17D | Interleukin-17D | Q8TAD2 |
| Interleukin-17E | Interleukin-25 | Q9H293 |
| Interleukin-17F | Interleukin-17F | Q96PD4 |
| Interleukin-18 binding protein | Interleukin-18-binding protein | O95998 |
| Interleukin-18 receptor α | Interleukin-18 receptor 1 | Q13478 |
| Interleukin-18 receptor β | Interleukin-18 receptor accessory protein | O95256 |
| Interleukin-19 | Interleukin-19 | Q9UHD0 |
| Interleukin-20 | Interleukin-20 | Q9NYY1 |
| Interleukin-22 | Interleukin-22 | Q9GZX6 |
| Interleukin-22 receptor α-1 | Interleukin-22 receptor subunit alpha-1 | Q8N6P7 |
| Interleukin-23 | Interleukin-23 | P29460, Q9NPF7 |
| Interleukin-24 | Interleukin-24 | Q13007 |
| Interleukin-27 | Interleukin 27 | Q8NEV9 |
| Interleukin-37 | Interleukin-1 family member 7 | Q9NZH6 |
| Junctional adhesion molecule B | Junctional adhesion molecule B | P57087 |
| Junctional adhesion molecule C | Junctional adhesion molecule C | Q9BX67 |
| Kallikrein 4 | Kallikrein-4 | Q9Y5K2 |
| Kallikrein 5 | Kallikrein-5 | Q9Y337 |
| Kallikrein 6 | Kallikrein-6 | Q92876 |
| Kallikrein 7 | Kallikrein-7 | P49862 |
| Kallikrein 8 | Neuropsin | O60259 |
| Kallikrein 11 | Kallikrein-11 | Q9UBX7 |
| Kallikrein 12 | Kallikrein-12 | Q9UKR0 |
| Kallikrein 13 | Kallikrein-13 | Q9UKR3 |
| Kallikrein 14 | Kallikrein-14 | Q9P0G3 |
| Kallistatin | Kallistatin | P29622 |
| Karyopherin-α2 | Importin subunit alpha-2 | P52292 |
| Kininogen, HMW, Single Chain | Kininogen-1 (single chain form) | P01042 |
| Kininogen, HMW, Two Chain | Kininogen-1 (two-chain form) | P01042 |
| Kremen protein 2 | Kremen protein 2 | Q8NCW0 |
| Kunitz-type protease inhibitor 2A | Kunitz-type protease inhibitor 2 | O43291 |
| Lactate dehydrogenase 1 (heart) | L-lactate dehydrogenase B chain | P07195 |
| Lactoferrin | Lactotransferrin | P02788 |
| Lamin-B1 | Lamin-B1 | P20700 |
| Laminin | Laminin subunits alpha-1, beta-1, and gamma-1 | P25391, P07942, P11047 |
| Langerin | C-type lectin domain family 4 member K | Q9UJ71 |
| Layilin | Layilin | Q6UX15 |
| LD78-β/CCL3L1 | Small-inducible cytokine A3-like 1 | P16619 |
| LEAP-1/Hepcidin | Hepcidin | P81172 |
| Legumain | Legumain | Q99538 |
| Leptin | Leptin | P41159 |
| Leucine-rich repeats and Ig-like domains protein 3 | Leucine-rich repeats and immunoglobulin-like domains protein 3 | Q6UXM1 |
| Leukotriene A-4 hydrolase | Leukotriene A-4 hydrolase | P09960 |
| Limbic system-associated membrane protein | Limbic system-associated membrane protein | Q13449 |
| Lipocalin 2 | Neutrophil gelatinase-associated lipocalin | P80188 |
| Lipopolysaccharide-binding protein | Lipopolysaccharide-binding protein | P18428 |
| Liver-expressed chemokine/CCL16 | Small-inducible cytokine A16 | O15467 |
| L-Selectin | L-selectin | P14151 |
| Luteinizing hormone | Glycoprotein hormones alpha chain and Lutropin subunit beta | P01215, P01229 |
| Lymphatic vessel endothelial hyaluronic acid receptor 1 | Lymphatic vessel endothelial hyaluronic acid receptor 1 | Q9Y5Y7 |
| Lymphocyte Activation Gene-1/CCL4L1 | Macrophage inflammatory protein-1b2 | Q8NHW4 |
| Lymphocyte antigen 86/Myeloid differentiation 1 | Lymphocyte antigen 86 | O95711 |
| Lymphotactin | Lymphotactin | P47992 |
| Lymphotoxin α1:β2 | Lymphotoxin-alpha (1) and Lymphotoxin-beta (2) | P01374, Q06643 |
| Lymphotoxin α2:β1 | Lymphotoxin-alpha (2) and Lymphotoxin-beta (1) | P01374, Q06643 |
| Lymphotoxin β receptor | Tumor necrosis factor receptor superfamily member 3 | P36941 |
| Lyn kinase | Tyrosine-protein kinase Lyn | P07948 |
| Lyn kinase, isoform B | Tyrosine-protein kinase Lyn, isoform B | P07948-2 |
| Lysozyme | Lysozyme C | P61626 |
| Macrophage colony-stimulating factor 1 | Macrophage colony-stimulating factor 1 | P09603 |
| Macrophage colony-stimulating factor 1 receptor | Macrophage colony-stimulating factor 1 receptor | P07333 |
| Macrophage inflammatory protein 1-α/CCL3 | C-C motif chemokine 3 | P10147 |
| Macrophage inflammatory protein 1-β/CCL4 | Small-inducible cytokine A4 | P13236 |
| Macrophage inflammatory protein 3 α/CCL20 | Small-inducible cytokine A20 | P78556 |
| Macrophage inflammatory protein 3 β/CCL19 | Small-inducible cytokine A19 | Q99731 |
| Macrophage inflammatory protein 4/Pulmonary and activation-regulated chemokine/CCL18 | C-C motif chemokine 18 | P55774 |
| Macrophage inflammatory protein 5/CCL15 | C-C motif chemokine 15 | Q16663 |
| Macrophage mannose receptor/MRC1 | Macrophage mannose receptor 1 | P22897 |
| Macrophage mannose receptor 2 | Macrophage mannose receptor 2 | Q9UBG0 |
| Macrophage scavenger receptor | Macrophage scavenger receptor types I and II | P21757 |
| Macrophage stimulatory protein receptor | Macrophage-stimulating protein receptor | Q04912 |
| Macrophage-derived chemokine | Small-inducible cytokine A22 | O00626 |
| Malate dehydrogenase, cytoplasmic | Malate dehydrogenase, cytoplasmic | P40925 |
| Mannan-binding lectin serine peptidase 1 | Complement-activating component of Ra-reactive factor splice variant MASP3 | P48740 |
| Mannose-binding protein C | Mannose-binding protein C | P11226 |
| MAPK 1 | Mitogen-activated protein kinase 1 | P28482 |
| MAPK 3/ERK-1 | Mitogen-activated protein kinase 3 | P27361 |
| MAPK 8 | Mitogen-activated protein kinase 8 | P45983 |
| MAPK 12 | Mitogen-activated protein kinase 12 | P53778 |
| MAPK 13 | Mitogen-activated protein kinase 13 | O15264 |
| MAPK 14 | Mitogen-activated protein kinase 14 | Q16539 |
| MAPK kinase 1 | Dual specificity mitogen-activated protein kinase kinase 1 | Q02750 |
| MAPK kinase 2 | Dual specificity mitogen-activated protein kinase kinase 2 | P36507 |
| MAPK-activated protein kinase 2 | MAP kinase-activated protein kinase 2 | P49137 |
| MAPK-activated protein kinase 3 | MAP kinase-activated protein kinase 3 | Q16644 |
| MAPKAPK5 | MAP kinase-activated protein kinase 5 | Q8IW41 |
| Marapsin | Serine protease 27 | Q9BQR3 |
| Matrilin-2 | Matrilin-2 | O00339 |
| Matrilin-3 | Matrilin-3 | O15232 |
| Matrix extracellular phosphoglycoprotein | Matrix extracellular phosphoglycoprotein | Q9NQ76 |
| Matrix metalloproteinase 2/Gelatinase A | 72 kDa type IV collagenase | P08253 |
| Matrix metalloproteinase 3/Stromelysin 1 | Stromelysin-1 | P08254 |
| Matrix metalloproteinase 7/Matrilysin/MMP-7 | Matrilysin | P09237 |
| Matrix metalloproteinase 8/Neutrophil collagenase | Neutrophil collagenase | P22894 |
| Matrix metalloproteinase 9/Gelatinase B | Matrix metalloproteinase-9 | P14780 |
| Matrix metalloproteinase 10/Stromelysin 2 | Stromelysin-2 | P09238 |
| Matrix metalloproteinase 12/Macrophage metalloelastase/MMP-12 | Macrophage metalloelastase | P39900 |
| Matrix metalloproteinase 17 | Matrix metalloproteinase-17 | Q9ULZ9 |
| MCM2 | DNA replication licensing factor MCM2 | P49736 |
| Mediator complex subunit 1 | Mediator of RNA polymerase II transcription subunit 1 | Q15648 |
| Megakaryocyte-associated tyrosine-protein kinase | Megakaryocyte-associated tyrosine-protein kinase | P42679 |
| Melanoma Inhibitory Activity | Melanoma-derived growth regulatory protein | Q16674 |
| Membrane frizzled-related protein | Membrane frizzled-related protein | Q9BY79 |
| Mesothelin | Mesothelin | Q13421 |
| Methionine aminopeptidase 1 | Methionine aminopeptidase 1 | P53582 |
| Methionine aminopeptidase 2 | Methionine aminopeptidase 2 | P50579 |
| Methyl-CpG-binding domain protein 4 | Methyl-CpG-binding domain protein 4 | O95243 |
| MHC class I chain-related protein A | MHC class I chain-related protein A | Q29983 |
| Microtubule-associated protein tau | Microtubule-associated protein tau | P10636 |
| Midkine | Midkine | P21741 |
| MIG | Small-inducible cytokine B9 | Q07325 |
| Monocyte chemoattractant protein 1 | Small-inducible cytokine A2 | P13500 |
| Monocyte chemoattractant protein 2 | Small-inducible cytokine A8 | P80075 |
| Monocyte chemoattractant protein 3 | Small-inducible cytokine A7 | P80098 |
| Monocyte chemoattractant protein 4 | Small-inducible cytokine A13 | Q99616 |
| Myeloid progenitor inhibitory factor 1/CCL23 | C-C motif chemokine 23 | P55773 |
| Myeloperoxidase | Myeloperoxidase | P05164 |
| Myoglobin | Myoglobin | P02144 |
| Myosin regulatory light chain 2 | Myosin regulatory light chain 2, ventricular/cardiac muscle isoform | P10916 |
| Myotonic dystrophy protein kinase-like β | Serine/threonine-protein kinase MRCK beta | Q9Y5S2 |
| N-acetyl-D-glucosamine kinase/NAGK | N-acetyl-D-glucosamine kinase | Q9UJ70 |
| N-acetylglucosamine-6-sulfatase | N-acetylglucosamine-6-sulfatase | P15586 |
| NADPH-P450 Oxidoreductase | NADPH--cytochrome P450 reductase | P16435 |
| Nascent polypeptide-associated complex α subunit | Nascent polypeptide-associated complex subunit alpha | Q13765 |
| Natural cytotoxicity triggering receptor 2 | Natural cytotoxicity triggering receptor 2 | O95944 |
| Natural cytotoxicity triggering receptor 3 | Natural cytotoxicity triggering receptor 3 | O14931 |
| Natural killer group 2 member D | NKG2-D type II integral membrane protein | P26718 |
| Nectin-like protein 1 | Cell adhesion molecule 3 | Q8N126 |
| Nectin-like protein 2 | Cell adhesion molecule 1 | Q9BY67 |
| Nephroblastoma Overexpressed gene homolog | Protein NOV homolog | P48745 |
| Neprilysin-2 | Membrane metallo-endopeptidase-like 1 | Q495T6 |
| Netrin-4 | Netrin-4 | Q9HB63 |
| Neural cell adhesion molecule 1, 120 kDa isoform | Neural cell adhesion molecule 1, 120 kDa isoform | P13591 |
| Neural cell adhesion molecule L1-like protein | Neural cell adhesion molecule L1-like protein | O00533 |
| Neuregulin-1 | Neuregulin-1 | Q02297 |
| Neuroblastoma suppressor of tumorigenicity 1 | Neuroblastoma suppressor of tumorigenicity 1 | P41271 |
| Neuropilin-1 | Neuropilin-1 | O14786 |
| Neurotrophic tyrosine kinase receptor type 1 | High affinity nerve growth factor receptor | P04629 |
| Neurotrophic tyrosine kinase receptor type 3 | NT-3 growth factor receptor | Q16288 |
| Neurotrophin-3 | Neurotrophin-3 | P20783 |
| Neurotrophin-5 | Neurotrophin-5 | P34130 |
| Neutral ceramidase | Neutral ceramidase | Q9NR71 |
| Neutrophil-activating peptide 2/NAP-2 | Neutrophil-activating peptide 2 | P02775 |
| Nidogen | Nidogen-1 | P14543 |
| Nidogen-2 | Nidogen-2 | Q14112 |
| Noggin | Noggin | Q13253 |
| Nogo Receptor | Reticulon-4 receptor | Q9BZR6 |
| Oncostatin M | Oncostatin-M | P13725 |
| Opioid-binding cell adhesion molecule | Opioid-binding protein/cell adhesion molecule | Q14982 |
| Osteoblast-specific transcription factor 2 | Runt-related transcription factor 2 | Q13950 |
| Osteonectin | SPARC | P09486 |
| Osteoprotegerin | Tumor necrosis factor receptor superfamily member 11B | O00300 |
| Osteoprotegerin ligand/TRANCE | Tumor necrosis factor ligand superfamily member 11 | O14788 |
| Otubain-1 | Ubiquitin thioesterase OTUB1 | Q96FW1 |
| Ovarian cancer immunoreactive antigen domain containing 1 | OCIA domain-containing protein 1 | Q9NX40 |
| OX40 Ligand/Tumor necrosis factor ligand superfamily member 4 | Tumor necrosis factor ligand superfamily member 4 | P23510 |
| Oxidized low-density lipoprotein receptor 1 | Oxidized low-density lipoprotein receptor 1 | P78380 |
| p21-activated kinase 3 | Serine/threonine-protein kinase PAK 3 | O75914 |
| p21-activated kinase 6 | Serine/threonine-protein kinase PAK 6 | Q9NQU5 |
| p21-activated kinase 7 | Serine/threonine-protein kinase PAK 7 | Q9P286 |
| Parathyroid hormone | Parathyroid hormone | P01270 |
| Parathyroid hormone-related protein | Parathyroid hormone-related protein | P12272 |
| Peptide YY | Peptide YY | P10082 |
| Peptidoglycan recognition protein, short | Peptidoglycan recognition protein | O75594 |
| Peroxiredoxin-1 | Peroxiredoxin-1 | Q06830 |
| Persephin | Persephin | O60542 |
| Phosphatase and tensin homolog | Phosphatidylinositol-3,4,5-trisphosphate 3-phosphatase and dual-specificity protein phosphatase PTEN | P60484 |
| Phosphatidylinositol-4,5-bisphosphate 3-kinase catalytic subunit γ isoform | Phosphatidylinositol-4,5-bisphosphate 3-kinase catalytic subunit gamma isoform | P48736 |
| Phosphoglycerate mutase 1 | Phosphoglycerate mutase 1 | P18669 |
| Phosphoinositide-3-kinase catalytic α polypeptide:regulatory subunit 1α complex | Phosphatidylinositol-4,5-bisphosphate 3-kinase catalytic subunit alpha isoform, Phosphatidylinositol 3-kinase regulatory subunit alpha Complex | P42336, P27986 |
| Phospholipase A2, Group IB | Phospholipase A2 | P04054 |
| Phospholipase A2, Group IIA | Phospholipase A2, membrane associated | P14555 |
| Phospholipase A2, Group IIE | Group IIE secretory phospholipase A2 | Q9NZK7 |
| Phospholipase A2, Group V | Calcium-dependent phospholipase A2 | P39877 |
| Phospholipase A2, Group X | Group 10 secretory phospholipase A2 | O15496 |
| Placenta growth factor | Placenta growth factor | P49763 |
| Plasmin | Plasmin heavy chain A and light chain B | P00747 |
| Plasminogen | Plasminogen | P00747 |
| Plasminogen activator inhibitor 1 | Plasminogen activator inhibitor 1 | P05121 |
| Platelet endothelial cell adhesion molecule | Platelet endothelial cell adhesion molecule | P16284 |
| Platelet factor 4 | Platelet factor 4 | P02776 |
| Platelet-activating factor acetylhydrolase IB subunit β/PAFAH β subunit | Platelet-activating factor acetylhydrolase IB subunit beta | P68402 |
| Platelet-derived growth factor A chain homodimer | Platelet-derived growth factor A chain | P04085 |
| Platelet-derived growth factor B chain homodimer | Platelet-derived growth factor B chain | P01127 |
| Platelet-derived growth factor C chain homodimer | Platelet-derived growth factor C chain | Q9NRA1 |
| Platelet-derived growth factor receptor β-type | Beta-type platelet-derived growth factor receptor | P09619 |
| Pleiotrophin | Pleiotrophin | P21246 |
| Polymeric immunoglobulin receptor | Polymeric immunoglobulin receptor | P01833 |
| Pregnancy-associated plasma protein-A | Pappalysin-1 | Q13219 |
| Prekallikrein | Plasma kallikrein (precursor) | P03952 |
| Programmed cell death 1 ligand 2 | Programmed cell death 1 ligand 2 | Q9BQ51 |
| Prolactin | Prolactin | P01236 |
| Proliferating cell nuclear antigen | Proliferating cell nuclear antigen | P12004 |
| Properdin | Properdin | P27918 |
| Prostate-specific membrane antigen | Glutamate carboxypeptidase 2 | Q04609 |
| Protease nexin I | Glia-derived nexin | P07093 |
| Proteasome subunit p40 | 26S proteasome non-ATPase regulatory subunit 7 | P51665 |
| Proteasome subunit α1 | Proteasome subunit alpha type-1 | P25786 |
| Proteasome subunit α6 | Proteasome subunit alpha type-6 | P60900 |
| Protein C | Vitamin K-dependent protein C | P04070 |
| Protein C Inhibitor | Plasma serine protease inhibitor | P05154 |
| Protein E7 (Human Papillomavirus Type 16) | Protein E7 (Human Papillomavirus Type 16) | P03129 |
| Protein E7 (Human Papillomavirus Type 18) | Protein E7 (Human Papillomavirus Type 18) | P06788 |
| Protein kinase B γ | RAC-gamma serine/threonine-protein kinase | Q9Y243 |
| Protein kinase C α | Protein kinase C alpha type | P17252 |
| Protein kinase C β type (splice variant β-II) | Protein kinase C beta type (splice variant Beta-II) | P05771 |
| Protein kinase C γ | Protein kinase C gamma type | P05129 |
| Protein kinase C δ | Protein kinase C delta type | Q05655 |
| Protein kinase C ζ | Protein kinase C zeta type | Q05513 |
| Protein kinase C θ | Protein kinase C theta type | Q04759 |
| Protein kinase C ι | Protein kinase C iota type | P41743 |
| Protein S | Vitamin K-dependent protein S | P07225 |
| Proteinase-3 | Myeloblastin | P24158 |
| Prothrombin | Prothrombin | P00734 |
| Proto-oncogene tyrosine-protein kinase FGR | Proto-oncogene tyrosine-protein kinase FGR | P09769 |
| Proto-oncogene tyrosine-protein kinase Fyn | Proto-oncogene tyrosine-protein kinase Fyn | P06241 |
| Proto-oncogene tyrosine-protein kinase LCK | Proto-oncogene tyrosine-protein kinase LCK | P06239 |
| Proto-oncogene tyrosine-protein kinase receptor Ret | Proto-oncogene tyrosine-protein kinase receptor ret | P07949 |
| Proto-oncogene tyrosine-protein kinase Src | V-src sarcoma (Schmidt-Ruppin A-2) viral oncogene homolog (Avian) | P12931 |
| Proto-oncogene tyrosine-protein kinase Yes | Proto-oncogene tyrosine-protein kinase Yes | P07947 |
| PSA | Prostate-specific antigen | P07288 |
| PSA:α-1-antichymotrypsin complex | Prostate-specific antigen and Alpha-1-antichymotrypsin | P07288, P01011 |
| P-Selectin | P-selectin | P16109 |
| Pyridoxal phosphate phosphatase | Pyridoxal phosphate phosphatase | Q96GD0 |
| Rab GDP dissociation inhibitor β | Rab GDP dissociation inhibitor beta | P50395 |
| RACK1 | Guanine nucleotide-binding protein subunit beta-2-like 1 | P63244 |
| RAC-α serine:threonine-protein kinase | RAC-alpha serine/threonine-protein kinase | P31749 |
| RAGE, soluble/Advanced glycosylation end product-specific receptor | Advanced glycosylation end product-specific receptor | Q15109 |
| RANTES/CCL5 | Small-inducible cytokine A5 | P13501 |
| Ras-related C3 botulinum toxin substrate 1 | Ras-related C3 botulinum toxin substrate 1 | P63000 |
| Receptor expressed in lymphoid tissues | Tumor necrosis factor receptor superfamily member 19L | Q969Z4 |
| Receptor-type tyrosine-protein kinase FLT3 | FL cytokine receptor | P36888 |
| Renin | Renin | P00797 |
| Repulsive guidance molecule A | Repulsive guidance molecule A | Q96B86 |
| Resistin | Resistin | Q9HD89 |
| Retinol-binding protein 4 | Retinol-binding protein 4 | P02753 |
| RGM domain family member B | RGM domain family member B | Q6NW40 |
| Ribosomal protein S3a | 40S ribosomal protein S3a | P61247 |
| Ribosomal protein S6 kinase 5 | Ribosomal protein S6 kinase alpha-5 | O75582 |
| Ribosomal protein S6 kinase α-3 | Ribosomal protein S6 kinase alpha-3 | P51812 |
| Ribosomal protein S7 | 40S ribosomal protein S7 | P62081 |
| Ribosome maturation protein SBDS | Ribosome maturation protein SBDS | Q9Y3A5 |
| RNA-binding motif protein 39 | RNA-binding protein 39 | Q14498 |
| S100A12 | Protein S100-A12 | P80511 |
| Scavenger receptor for phosphatidylserine and oxidized low density lipoprotein/CXCL16 | Small-inducible cytokine B16 | Q9H2A7 |
| Secretin | Secretin | P09683 |
| Secretory leukocyte protease inhibitor/SLPI | Antileukoproteinase | P03973 |
| Seizure 6-like protein 2 | Seizure 6-like protein 2 | Q6UXD5 |
| Semaphorin 3A | Semaphorin-3A | Q14563 |
| Serine-threonine-protein kinase 16 | Serine/threonine-protein kinase 16 | O75716 |
| Serine-threonine-protein kinase Chk1 | Serine/threonine-protein kinase Chk1 | O14757 |
| Serine-threonine-protein kinase Chk2 | Serine/threonine-protein kinase Chk2 | O96017 |
| Serine-threonine-protein kinase PLK1 | Serine/threonine-protein kinase PLK1 | P53350 |
| Serum amyloid A | Serum amyloid A protein | P02735 |
| Serum amyloid P | Serum amyloid P-component | P02743 |
| SET9 | Histone-lysine N-methyltransferase, H3 lysine-4 specific SET7 | Q8WTS6 |
| Siglec-3 | Myeloid cell surface antigen CD33 | P20138 |
| Siglec-6 | Sialic acid-binding Ig-like lectin 6 | O43699 |
| Siglec-7 | Sialic acid-binding Ig-like lectin 7 | Q9Y286 |
| Siglec-9 | Sialic acid-binding Ig-like lectin 9 | Q9Y336 |
| Signaling lymphocytic activation molecule 5 | SLAM family member 5 | Q9UIB8 |
| SLIT and NTRK-like protein 1 | SLIT and NTRK-like protein 1 | Q96PX8 |
| Small glutamine-rich tetratricopeptide repeat-containing protein α | Small glutamine-rich tetratricopeptide repeat-containing protein alpha | O43765 |
| Soggy-1 | Dickkopf-like protein 1 | Q9UK85 |
| Somatostatin-28 | Somatostatin-28 | P61278 |
| Sonic Hedgehog | Sonic hedgehog protein | Q15465 |
| Sorting nexin 4 | Sorting nexin-4 | O95219 |
| S-phase kinase-associated protein 1 | S-phase kinase-associated protein 1 | P63208 |
| Stabilin-2 | Stabilin-2 | Q8WWQ8 |
| Stem cell factor receptor/CD117/c-Kit | Mast/stem cell growth factor receptor | P10721 |
| Stem Cell Growth Factor-α | C-type lectin domain family 11 member A (alpha form) | Q9Y240 |
| Stem Cell Growth Factor-β | C-type lectin domain family 11 member A (beta form) | Q9Y240 |
| Stress-induced-phosphoprotein 1 | Stress-induced-phosphoprotein 1 | P31948 |
| Stromal cell-derived factor 1α | SDF-1-alpha | P48061 |
| Stromal cell-derived factor 1β | SDF-1-beta | P48061 |
| SUMO-conjugating enzyme UBC9 | SUMO-conjugating enzyme UBC9 | P63279 |
| Superoxide dismutase [Cu-Zn] | Superoxide dismutase [Cu-Zn] | P00441 |
| Survivin | Baculoviral IAP repeat-containing protein 5 | O15392 |
| Syntaxin 1A | Syntaxin-1A | Q16623 |
| TANK-binding kinase 1 | Serine/threonine-protein kinase TBK1 | Q9UHD2 |
| Tartrate-resistant acid phosphatase type 5/TrATPase | Tartrate-resistant acid phosphatase type 5 | P13686 |
| TATA-box-binding protein | TATA-box-binding protein | P20226 |
| T-cell surface glycoprotein CD4 | T-cell surface glycoprotein CD4 | P01730 |
| Tenascin | Tenascin | P24821 |
| Testican-1 | Testican-1 | Q08629 |
| Testican-2 | Testican-2 | Q92563 |
| Thrombin | Thrombin heavy and light chains | P00734 |
| Thrombin-Activatable Fibrinolysis Inhibitor | Carboxypeptidase B2 | Q96IY4 |
| Thrombopoietin | Thrombopoietin | P40225 |
| Thrombopoietin Receptor | Thrombopoietin receptor | P40238 |
| Thrombospondin-1 | Thrombospondin-1 | P07996 |
| Thrombospondin-2 | Thrombospondin-2 | P35442 |
| Thrombospondin-4 | Thrombospondin-4 | P35443 |
| Thymic stromal lymphopoietin | Thymic stromal lymphopoietin | Q969D9 |
| Thymic stromal lymphopoietin protein receptor | Thymic stromal lymphopoietin protein receptor | Q9HC73 |
| Thymus and activation-regulated chemokine/CCL17 | Small-inducible cytokine A17 | Q92583 |
| Thymus expressed chemokine/CCL25 | Small-inducible cytokine A25 | O15444 |
| Thyroglobulin | Thyroglobulin | P01266 |
| Thyroid peroxidase | Thyroid peroxidase | P07202 |
| Thyroid Stimulating Hormone | Glycoprotein hormones alpha and Thyrotropin subunit beta chains | P01215, P01222 |
| Thyroxine-Binding Globulin | Thyroxine-binding globulin | P05543 |
| Tissue factor pathway inhibitor | Tissue factor pathway inhibitor | P10646 |
| Tissue inhibitor of metalloproteinases 1 | Metalloproteinase inhibitor 1 | P01033 |
| Tissue inhibitor of metalloproteinases 2 | Metalloproteinase inhibitor 2 | P16035 |
| Tissue inhibitor of metalloproteinases 3 | Metalloproteinase inhibitor 3 | P35625 |
| Tissue-type plasminogen activator | Tissue-type plasminogen activator | P00750 |
| T-lymphocyte activation antigen CD80 | T-lymphocyte activation antigen CD80 | P33681 |
| T-lymphocyte surface antigen Ly-9/CD229 | T-lymphocyte surface antigen Ly-9 | Q9HBG7 |
| Toll-like receptor 2 | Toll-like receptor 2 | O60603 |
| Toll-like receptor 4 | Toll-like receptor 4 | O00206 |
| Topoisomerase I | DNA topoisomerase 1 | P11387 |
| Transferrin | Serotransferrin | P02787 |
| Transforming growth factor β induced protein | Transforming growth factor-beta-induced protein ig-h3 | Q15582 |
| Transforming growth factor β receptor type III | TGF-beta receptor type III | Q03167 |
| Transforming growth factor β-1 | Transforming growth factor beta-1 | P01137 |
| Transforming growth factor β-2 | Transforming growth factor beta-2 | P61812 |
| Transforming growth factor β-3 | Transforming growth factor beta-3 | P10600 |
| Troponin I | Troponin I, cardiac muscle | P19429 |
| Troponin T | Troponin T, cardiac muscle | P45379 |
| Trypsin | Trypsin-1 | P07477 |
| Trypsin-3 | Trypsin-3 | P35030 |
| Tryptase β-2 | Tryptase beta-2 | P20231 |
| Tryptase γ | Tryptase gamma | Q9NRR2 |
| Tumor necrosis factor ligand superfamily member 12 | Tumor necrosis factor ligand superfamily member 12 | O43508 |
| Tumor necrosis factor ligand superfamily member 15 | Tumor necrosis factor ligand superfamily member 15 | O95150 |
| Tumor necrosis factor ligand superfamily member 18 | Tumor necrosis factor ligand superfamily member 18 | Q9UNG2 |
| Tumor necrosis factor receptor superfamily member 1A | Tumor necrosis factor receptor superfamily member 1A | P19438 |
| Tumor necrosis factor receptor superfamily member 1B | Tumor necrosis factor receptor superfamily member 1B | P20333 |
| Tumor necrosis factor receptor superfamily member 4 | Tumor necrosis factor receptor superfamily member 4 | P43489 |
| Tumor necrosis factor receptor superfamily member 10D | Tumor necrosis factor receptor superfamily member 10D | Q9UBN6 |
| Tumor necrosis factor receptor superfamily member 13B | Tumor necrosis factor receptor superfamily member 13B | O14836 |
| Tumor-associated calcium signal transducer 2 | Tumor-associated calcium signal transducer 2 | P09758 |
| Tyrosine kinase Bruton | Tyrosine-protein kinase BTK | Q06187 |
| Tyrosine kinase Etk | Cytoplasmic tyrosine-protein kinase BMX | P51813 |
| Tyrosine-protein kinase 6 | Tyrosine-protein kinase 6 | Q13882 |
| Tyrosine-protein kinase receptor Tie-1, soluble | Tyrosine-protein kinase receptor Tie-1 | P35590 |
| Tyrosine-protein kinase receptor Tie-2, soluble/Angiopoietin-1 receptor | Angiopoietin-1 receptor | Q02763 |
| Tyrosine-protein kinase receptor TYRO3 | Tyrosine-protein kinase receptor TYRO3 | Q06418 |
| Tyrosine-protein kinase Tec | Tyrosine-protein kinase Tec | P42680 |
| Tyrosine-protein kinase transmembrane receptor ROR1 | Tyrosine-protein kinase transmembrane receptor ROR1 | Q01973 |
| Tyrosine-protein phosphatase non-receptor type 1 | Tyrosine-protein phosphatase non-receptor type 1 | P18031 |
| Tyrosine-protein phosphatase non-receptor type 11 | Tyrosine-protein phosphatase non-receptor type 11 | Q06124 |
| Tyrosine-protein phosphatase non-receptor type 2 | Tyrosine-protein phosphatase non-receptor type 2 | P17706 |
| Ubiquitin | Ubiquitin | P62979 |
| Ubiquitin-conjugating enzyme E2 L3 | Ubiquitin-conjugating enzyme E2 L3 | P68036 |
| Ubiquitin-conjugating enzyme E2 N | Ubiquitin-conjugating enzyme E2 N | P61088 |
| Ubiquitin-fold modifier 1 | Ubiquitin-fold modifier 1 | P61960 |
| Ubiquitin-fold modifier-conjugating enzyme 1 | Ufm1-conjugating enzyme 1 | Q9Y3C8 |
| UL16 binding protein 3 | NKG2D ligand 3 | Q9BZM4 |
| UL16-binding protein 1/NKG2D ligand 1 | NKG2D ligand 1 | Q9BZM6 |
| UL16-binding protein 2/NKG2D ligand 2 | NKG2D ligand 2 | Q9BZM5 |
| Urokinase plasminogen activator surface receptor | Urokinase plasminogen activator surface receptor | Q03405 |
| Urokinase-type plasminogen activator/uPA | Urokinase-type plasminogen activator | P00749 |
| v-abl Abelson murine leukemia viral oncogene homolog 2 | Tyrosine-protein kinase ABL2 | P42684 |
| Vaccinia Virus VH1-related Phosphatase/Dual specificity protein phosphatase 3 | Dual specificity protein phosphatase 3 | P51452 |
| Vascular cell adhesion protein 1 | Vascular cell adhesion protein 1 | P19320 |
| Vascular endothelial growth factor A/VEGF | Vascular endothelial growth factor A | P15692 |
| Vascular endothelial growth factor C | Vascular endothelial growth factor C | P49767 |
| Vascular endothelial growth factor D | Vascular endothelial growth factor D | O43915 |
| Vascular endothelial growth factor receptor 2 | Vascular endothelial growth factor receptor 2 | P35968 |
| Vascular endothelial growth factor receptor 3 | Vascular endothelial growth factor receptor 3 | P35916 |
| Vasoactive Intestinal Peptide | Vasoactive intestinal peptide | P01282 |
| von Willebrand factor/vWF | von Willebrand factor | P04275 |
| WNK3 | Serine/threonine-protein kinase WNK3 | Q9BYP7 |
| Wnt inhibitory factor 1 | Wnt inhibitory factor 1 | Q9Y5W5 |
| WNT1-inducible-signaling pathway protein 1 | WNT1-inducible-signaling pathway protein 1 | O95388 |
| WNT1-inducible-signaling pathway protein 3 | WNT1-inducible-signaling pathway protein 3 | O95389 |
| X-linked ectodysplasin-A2 receptor | Tumor necrosis factor receptor superfamily member 27 | Q9HAV5 |
| X-Pro aminopeptidase 1 | Xaa-Pro aminopeptidase 1 | Q9NQW7 |
| YKL-40/Chitinase-3-like protein 1 | Chitinase-3-like protein 1 | P36222 |
| ZAP70/70 kDa zeta-associated protein kinase | Tyrosine-protein kinase ZAP-70 | P43403 |
| These names represent the protein targets against which the SOMAmers were selected. Specificity determinations have not been made on the entire menu. | |  |
